# Preclinical optimization of a broad-spectrum anti-bladder cancer tri-drug regimen *via* the Feedback System Control (FSC) platform

**DOI:** 10.1038/srep11464

**Published:** 2015-06-19

**Authors:** Qi Liu, Cheng Zhang, Xianting Ding, Hui Deng, Daming Zhang, Wei Cui, Hongwei Xu, Yingwei Wang, Wanhai Xu, Lei Lv, Hongyu Zhang, Yinghua He, Qiong Wu, Moshe Szyf, Chih-Ming Ho, Jingde Zhu

**Affiliations:** 1 School of Life Science and Technology, State Key Laboratory of Urban Water Resource and Environment, Harbin Institute of Technology, Harbin, Heilongjiang 150001, China, and Department of Anatomy and Cell Biology, University of Iowa, Carver College of Medicine, Iowa City, IA 52242, USA; 2 Department of Urology, The First Affiliated Hospital of Harbin Medical University, Harbin, Heilongjiang Province, China; 3 Med-X Research Institute, School of Biomedical Engineering, Shanghai Jiao Tong University, Shanghai, 200030, China; 4 Cancer Epigenetics Program, Anhui Cancer Hospital, Hefei, Anhui 230031, China; 5 Department of Neurosurgery, The First Affiliated Hospital of Harbin Medical University, Harbin, Heilongjiang Province, China; 6 Department of Pathology, The First Affiliated Hospital of Harbin Medical University, Harbin, Heilongjiang Province, China; 7 Department of Urology, The Forth Affiliated Hospital of Harbin Medical University, Harbin, Heilongjiang Province, China; 8 Cancer Epigenetics Program, Shanghai Cancer Institute, Renji Hospital, Shanghai Jiaotong University, Shanghai 200032, China; 9 Department of Pharmacology and Therapeutics McGill University Medical School 3655 Sir William Osler Promenade #1309, Montreal, Quebec Canada; 10 Mechanical and Aerospace Engineering Department, Biomedical Engineering Department, University of California, Los Angeles, CA 90095-1597, USA

## Abstract

Therapeutic outcomes of combination chemotherapy have not significantly advanced during the past decades. This has been attributed to the formidable challenges of optimizing drug combinations. Testing a matrix of all possible combinations of doses and agents in a single cell line is unfeasible due to the virtually infinite number of possibilities. We utilized the Feedback System Control (FSC) platform, a phenotype oriented approach to test 100 options among 15,625 possible combinations in four rounds of assaying to identify an optimal tri-drug combination in eight distinct chemoresistant bladder cancer cell lines. This combination killed between 82.86% and 99.52% of BCa cells, but only 47.47% of the immortalized benign bladder epithelial cells. Preclinical *in vivo* verification revealed its markedly enhanced anti-tumor efficacy as compared to its bi- or mono-drug components in cell line-derived tumor xenografts. The collective response of these pathways to component drugs was both cell type- and drug type specific. However, the entire spectrum of pathways triggered by the tri-drug regimen was similar in all four cancer cell lines, explaining its broad spectrum killing of BCa lines, which did not occur with its component drugs. Our findings here suggest that the FSC platform holdspromise for optimization of anti-cancer combination chemotherapy.

Although there have been significant advances in our understanding of the molecular basis of cancer and several hundred-targeted therapeutics were introduced based on these discoveries, chemotherapeutic regimens that are the mainstay of cancer treatment remain largely unchanged^1^. Most anticancer drugs have narrow therapeutic indices, leading to suboptimal dosing, treatment delay, or discontinuance and reduced patient compliance to therapy^2^. The idea of combination chemotherapy, also known as multicomponent therapies^3^, using two or more drugs that have no overlapping anti-cancer activities and systemic toxicities was first introduced in the late 1970’s^4^. This approach has improved the cure rate for Hodgkin’s lymphoma from 20 to80% and for lymph sarcoma from 15% to over 50%^4^,^5^. Since then, combination chemotherapy has gradually replaced single drug therapy in cancer^5^. Nevertheless, improvements to chemotherapy in the last five decades have been slow^6^. One of the key causes is that the current combination chemotherapy regimens are often derived from retrospective analyses of clinical trials^7^,^8^,^9^ and cell culture-based assays with an inadequate capacity to assess all possible combinations that vary in the number, type, and doses of drugs, while simultaneously optimizing for multiple conditions (e.g. efficacy and safety)^8^,^10^. Cell based optimization efforts assisted by mathematical methods were introduced in the late 1990 s^11^,^12^. Additional approaches include the classical is obologram method^13^, “envelope of additivity” method to distinguish cytotoxic agents that do not significantly interact^14^, and the Median effect analysis method introduced by Chou and Talalay^15^,^16^. One limitation of all current methods is that they are limited to bi-drug interactions, despite the fact that the majority of the combination regimens used in clinics today involve three or more drugs.

An obvious but prohibitive approach is the testing of all possible combinations of all drugs at all doses for the best regimen of the markedly improved therapeutic index. However, an effort of this kind exceeds the screening capacity of today’s biomedical research laboratories. Moreover, the extensive heterogeneity at the genetic, epigenetic, expressional, and phenotypic levels of cancer cells in patients necessitates testing a large number of cancer cell lines in order to represent disease diversity, which further amplifies the task.

Bladder cancer (BCa) is the fourth most common type of tumors in males worldwide^17^. Notorious for its recurrence and refractoriness to chemotherapy, BCa is one of the most difficult and costly malignancies^18^. Treatments for muscle-invasive bladder cancer have not advanced beyond cisplatin-centered combination chemotherapy and surgery in the past 30 years^1^.Median survival for patients with recurrent or metastatic bladder cancer remains at 14–15 months^19^,^20^. A recent multi-omic analysis of 131 bladder cancer patient samples produced a comprehensive picture of the genetic defects and expression abnormalities associated with BCa^21^, but few clues were offered for better diagnostic and therapeutic opportunities. Pathologically, bladder cancer consists of two major types: transitional cell carcinoma (TCC) accounting for more than 90% and squamous cell carcinoma for 6% to 8% of cases. There were earlier attempts to develop algorithms, such as BTSC and MOTSC to assist the experimental optimization of the combination therapies^3^,^22^,^23^.In this study, we used the Feedback System Control (FSC) platform, as a search algorithm (a differential evolution (DE) algorithm)^24^,^25^ (Fig. 1) and we derived effective combinations by testing less than 1% of all the possible combinations. The FSC platform focuses on a definable phenotypic outcome, such as drug-triggered cell death as in this study, rather than on detailed mechanistic characteristics. By harnessing the mechanism-independent and multi-parametric optimization capabilities of the FSC platform, we have previously successfully identified optimal drug combinations for viral infection inhibition, herpes virus reactivation, and the growth factor component regimen for human ES cells^24^,^25^,^26^,^27^.

In this study, we used this platform to quickly identify an effective tri-drug combination that is capable of killing seven TCC cell lines and one squamous cancer cell line that better represent the clinical spectrum of bladder cancer than any studies using a single or fewer cell lines. In contrast, this regimen possesses a significantly lower killing capability on immortalized benign epithelial cells, an indication that its broad anti-cancer cytotoxicity. It also more effectively suppressed the *in vivo* growth of three BCa-cell-line-derived-tumor-xenografts in nude mice than did its mono- or bi-drug constituents. By determining the activities of nine cancer-associated pathways in four bladder cancer cell lines and one immortalized normal cell line, we showed that the collective response of these pathways to the single component drug of the tri-drug combination is both drug type and cell line-specific. However, the entire spectrum of pathways activated by the tri-drug regimen was similar in all four cancer cell lines. This explains why the tri-drug combination has a broad anti-cancer effect but its component drugs fail. The broad applicability of FSC for the optimal drug combinations to a wide range of disease indications is fully anticipated.

## Results

### Bladder cancer cell lines vary considerably in their chemoresistance properties

**Figure 1:**
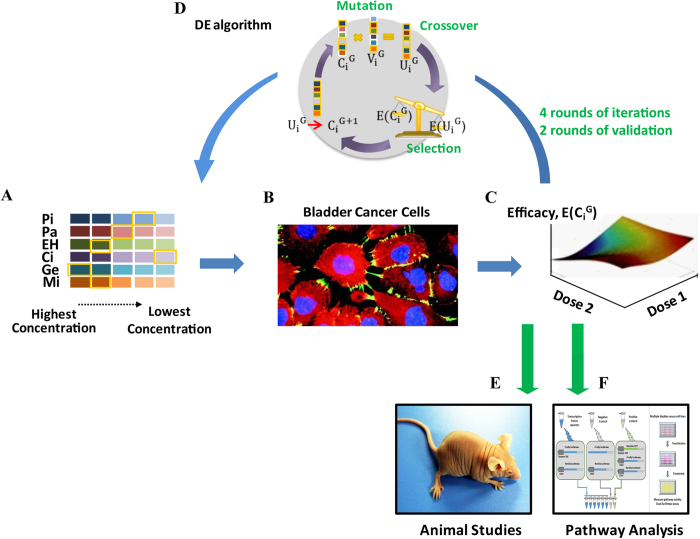
The scheme of study. **(A)** The drug-dose pool suggested from the chemoresistance profiling, consisted of six drugs at five doses. The multi-drug combinations were generated by the “Differential Evolution” (DE) algorithm from a matrix of six different drugs at five different doses. **(B)** The cells were subjected to treatment with the multi-drug combinations. The accumulated cell survival (ACS) was derived from the combined cell survival (%) of each cell line under the drug treatment. g combinations in each round of testing (iteration) were fed into the FSC scheme to generate the combinations for the subsequent round of iterations. **(C)** Measuring of the efficacy of the combinations. **(D)** Guided by the differential evolution algorithm deriving new drug-dose combination. Repeat B, C and D, until the optimal drug-dose combination was reached. The statistical modeling of the experimental data was generated by 4 rounds of iterations, **v**alidation (2 rounds). **(E)** The tumor inhibition efficacy of the EH/Ci/Mi regimen was compared with its bi- and mono-drug counterparts in the three-cell line-derived tumor xenograft/nude mice systems. Photo of the nude mouse was taken by co-author J.Z. **(F)** Pathway analysis, the activity of sixteen chemo-resistance associated pathways was determined under base line or the drug treated EH/Ci/Mi regimen or the single drug at four fold dose in the following four bladder cancer cell lines: 5637, Biu87, UM-UC-3, H-bc, and the normal cell line: SV-HUC-1.

**Figure 2:**
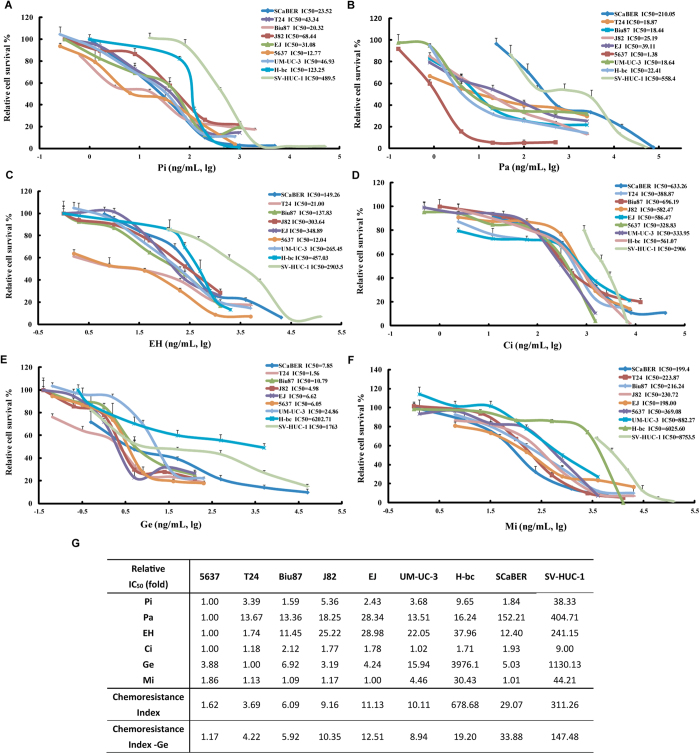
The chemoresistance profile of bladder cancer and immortalized untransformed cell lines. The relative cell survival (%) of the drug treated over the no drug treated cells (Y-axis) was plotted against the dose (ng/mL, lg) of the following six drugs (X-axis): Pi, Pa, EH, Ge, Ci, and Mi, respectively, and the curves were plotted for SCaBER, T24, Biu87, J82, EJ, 5637, UM-UC-3, H-bc, and SV-HUC-1 cell lines, respectively (panel **A**–**F**). **(G)** The relative IC_50_ (fold) of each drug with the lowest IC_50_ (ng/mL, lg) as reference was calculated.

We used the MTT (Test by thiazolyl blue tetrazolium blue) based assay to determine the dose required for 50% of cells to be killed (IC50) by following six anti-bladder cancer drugs (https://www.nccn.org/store/login/login.aspx? ReturnURL =  http://www.nccn.org/professionals/physician_gls/pdf/bladder.pdf):Pirarubicin (Pi), Paclitaxel (Pa), Epirubicin Hydrochloride (EH), Cisplatin (Ci), Gemcitabine (Ge), and Mitomycin (Mi) in eight human bladder cancer cell lines (7 TCC lines: T24, Biu87, EJ, J82, UM-UC-3, 5637, and H-bc and one squamous carcinoma cell line (SCaBER) and an immortalized benign cell line (SV-HUC-1) (Fig. 2A–F). The relative IC50 (fold) of each drug of all nine cell lines was normalized to the lowest IC50 in the most sensitive cell line (Fig. 2G), to describe the single drug resistance of cell lines. The overall chemoresistance of each cell line, a “chemoresistance index” (the numerator of the total relative IC50 over the number of drugs) (Fig. 2G) was calculated to rank the overall resistance state of each cell line to these six chemotherapeutics. The results from the IC50 profiling of these nine cell lines (Fig. 1) illustrated the considerable heterogeneity in drug resistance among BCa cell lines, despite an exception: an almost uniform sensitivity of all cell lines to Cisplatin. 5637 exhibited the highest chemo-sensitivity to four of six drugs and overall chemo-sensitivity for all six drugs (“chemoresistance” index: 1.62). H-bc was the most chemoresistant to three of six drugs, with an exceedingly high relative IC50 to GE (3976.1), which makes it the most chemoresistant (thechemoresistance index: 678.68). It was the second highest in the cancer cell line (thechemoresistance index: 19.2) when GE was taken out of consideration (Fig. 2G), to SCaBER (“chemoresistance index”: 33.88). SV-HUC-1, an immortalized benign epithelial cell line exhibited the highest IC50 to each of these five drugs and therefore the highest chemoresistance index: 147.28, which is almost 3.5 fold higher than SCaBER’s (Fig. 2G). Therefore, these five drugs have a high level of selective cancer cell killing capability.

### Identification of an effective tri-drug combination by Differential Evolution (DE) algorithm guided experimental screening (the FSC platform)

**Figure 3:**
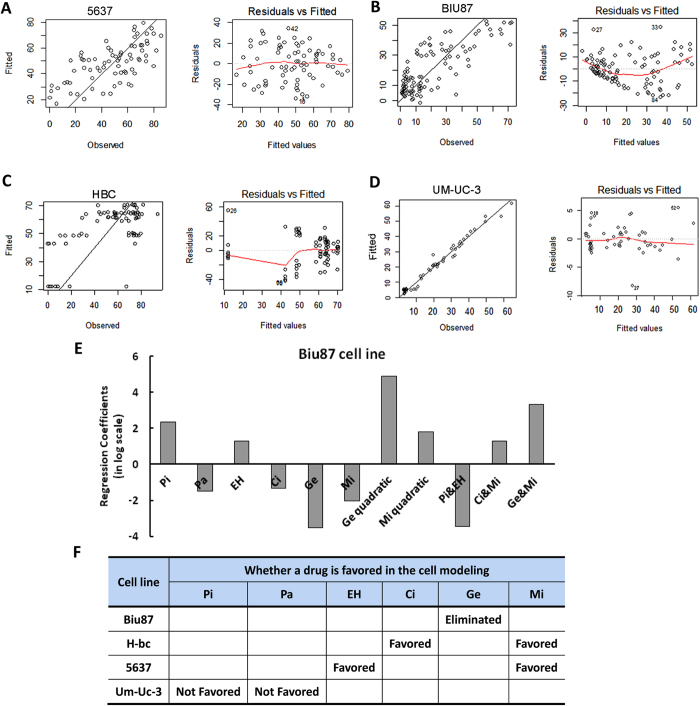
Statistic modeling analysis. Both experimental data from the DE algorithm guided testing and the model-predicted results of (**A**) 5637 (**B**) Biu87 (**C**) H-bc, and (**D**) UM-UC-3 cell lines are plotted together for comparison. (**E**) The Linear regression model analysis of the results in Biu8 cell line indicated that Ge was not a favored drug for combination and was not further used. (**F**) The summary of the combination ability of each pair drugs from the statistic modeling analysis.

The total number of possible combinations for six drugs at five doses is 15,625 for one cell line and 125,000 for all eight BCa cell lines. This represents a prohibitive scale of workload if all potential combinations are examined. To experimentally identify an effective tri-drug combination in this study, the DE algorithm version of the FSC platform was implemented (for further description see Supplementary material 2 and Fig. S1). The FSC platform consists of 4 iterative operations: 1) starting with a randomly selected drug-dose combination, 2) experimental assessment of combination-mediated cell killing efficacy, 3) optimization of the combinations via the DE algorithm, and 4) completion of the feedback control loop by testing the newly selected drug-dose combination until an optimum is reached (Fig. 3).

Twenty six-drug combinations at the indicated doses were initially generated through a random number generator (using MATLAB MathWorks©, Natick, MA, U.S.) (The upper half of Table 1). The cell survival (% over the no drug control) of treated cells was determined and then used for a calculation of the average cumulative cell survival (%, ACS: the sum of cell survival (%) per combination divided by the number of cell lines tested). ACS < 30% was used to separate the effective from the not effective combinations. Nine of the twenty combinations tested in the 1^st^ round of iteration fell into this category (the lower half of Table 1).

The ACS of each combination of the 1^st^ round testing was fed into a DE algorithm scheme to generate a new list of combinations for the next round of testing. Four (4) of the 20six-drug combinations tested in the 2^nd^ round of iteration were effective (Table S2). To reduce the number of drugs in combinations from six to four or five, a zero dose option was introduced in the 3^rd^and the 4^th^ round of testing for four or five drug combinations. The number of the effective combinations was raised from the 2^nd^ round (25%, 4 out of 20) (Table S2) to the 3^rd^ round (45%, 9 of 20) (Table S3), and the 4^th^ round (82.5%, 33 out of 40) of testing (Table 2), demonstrating improved optimization with the “evolution” of drug combinations (Fig. S2). More specifically, combination #40 (EH 250 ng/mL, highest dose; Ci, and 1500 ng/mL highest dose; Mi, and 200 ng/mL at the second highest dose, and Pi at the lowest dose, 0.32 ng/mL) produced the lowest ACS (13.60%) (Table 2). Pi, in this combination was at the lowest concentration and did not have any apparent impact on maintaining the efficient cell killing abilities of this regimen, as suggested by a comparative analysis with other Pi inclusive combinations (Table 1,2, Table S2-3). Therefore, we removed Pi and selected the combination 40 minus Pi (EH/Ci/Mi) as the candidate combination for further refinement.

### Statistical modeling analysis confirmed EH/Ci/M as the optimal tri-drug combination

For additional support, we performed statistical modeling^28^ analysis of all the features of the 100 combinations (Tables 1, 2 and S1-2) from testing of the following four cell lines: 5637 (sensitive), H-bc and UM-UC-3 (resistant), and Biu87 (moderately resistant) (Fig. 3).

For a given cancer cell line treated by six (k) different drugs (Pi, Pa, EH, Ci, Ge, and Mi), the system output, y, can be expressed by a 2^nd^ order polynomial regression model of drug doses x_i_. The data confirm that higher than 2^nd^ order terms do not make significant contributions to the efficacy, as was previously demonstrated in a lung cancer model^29^.


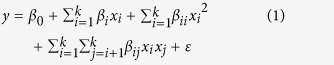


where 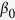, 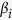, 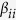 and 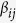 are the intercept, linear, quadratic, and bilinear (or interaction) terms^30^. The “observed” values represent the experimental data (ACS) and the “fitted” results represent the model predictions for the same drug combinations. A 2^nd^ order polynomial regression model fits the experimental results of Biu87, 5637, UM-UC-3, and H-bc which were plotted for comparison (Fig. 3).

We further examined the regression coefficients in the statistical modeling for each of these four cell lines. For instance, the polynomial regression model for Biu87 is:


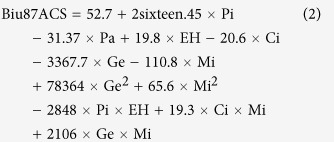


where Biu87ACS represents the ACS for Biu87 cells under different drug treatments and Pi, Pa, EH, Ci, Ge, and Mi in the equation (2) represent the absolute dosages of each drug (ng/mL). The model has a R^2^ value of 0.7015 and p-value equal to 2.439e-15, which indicates a good fit between the model and experimental observations. The chemotherapeutic agent Ge has a negative linear regression coefficient but the largest positive quadratic regression coefficient. This implies that a dose increase of Ge is less likely to enhance drug-triggered cell death when it is used in combination with other drugs. Ge also has the largest positive two-drug interaction coefficient with Mi, implying that Ge and Mi would have antagonistic effects in reducing cell survival. Therefore, we eliminated Ge from further consideration.

The regression model for the H-bc cell line is:


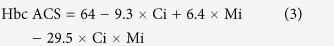


For the H-bc cell line, only Ci and Mi are left in the final regression model and the rest of the drugs do not appear in the model. This equation (3) instructs us that Ci and Mi are the most potent cytotoxic drugs for H-bc, which is resistant to other drugs. The interaction coefficient of C_i_ × M_i_ is negative, suggesting a synergistic cytotoxic effect of these two drugs on the H-bc cell line (for a compatible analysis, see Supplementary material 3).

In summary, the statistical modeling analysis supported the conclusion that the EH/Ci/Mi regimen is the optimized regimen as suggested by testing 100 of 15,625 possible combination for a single cell line *via* DE algorithm guided experimental testing.

### The EH/Ci/Mi regimen more effectively and selectively kills cancer cell lines than the component drugs at four fold doses

For further confirmation, the ACS of 34 combinations where both Ci and Mi were fixed at one of the two doses (Ci = 1500 and Mi = 1000 ng/mL), or the second highest doses (Ci = 300 and Mi = 200 ng/mL) alone or with one of the third drugs at one of three doses (the upper half of Table 3) were determined in the following TCC cell lines: Biu87, T24, 5637, UM-UC-3, and H-bc. Again, the EH/Ci/Mi regimen (1500 ng/mL Ci, 200 ng/mL Mi and 250 ng/mL EH,) showed the lowest ACS value (7.97%) among all of the four tri-drug regimens (combinations 11–14) which was finally examined in all eight BCa cell lines (Table S3). The EH/Ci/Mi regimen (ACS: 6.32%, Table S4) not only delivered more cell killing than other tri-drug regimens: Pi/Ci/Mi and Pa/Ci/Mi (Table S4), but was also more effective than the component drugs at four fold doses: Ci (6000 ng/mL, ACS: 16.60% ± 13.53%), Mi (800 ng/mL, ACS: 40.49% ± 21.63%) or EH (1000 ng/mL, ACS: 9.11% ± 7.29%), respectively (or Pi, 160 ng/ml, 21.32 ± 12.55%, or Pa, 640 ng/ml, 20.89 ± 12.73% Fig. S4A). This observation suggests a synergistic action of the three drugs when they are used in combination. Furthermore, the EH/Ci/Mi regimen possesses a broad spectrum of anti-bladder cancer activity, by killing between 82.86% and 99.52% of BCa cells in comparison to only 47.47% of SV-HUC-1 cells (Table S4). However, Ci is the most toxic to the immortalized normal cell line (SV-HUC-1), resulting in 73.79% cell death in comparison with EH (38.11%), Mi (8.83%), Pi (8.21%), Pa (19.83%), and EH/Ci/Mi (52.53%) cell death. Taking both the anti-BCa cell line (ACS%) and toxicity on the immortalized epithelial cells (Cell survival %) into account, we created the therapeutic window (TW) (Table S4 and Fig. S3), referring the difference (%) in the treatment trigger cell survival between SV-HUC-1 and BCa cell lines and the therapeutic window index (TW index), referring to the difference (%) between the therapeutic window and the ACS % to rank the EH/CI/Mi regimen and three component drug at a threefold higher dose (Table S4 and Fig. S3). The EH/CI/Mi regimen has the most favorable TW index (34.83%) than all four fold doses of each component drugs.

### The basal and drug-triggered levels of nine cancer-associated signaling pathway activities underlie the BCa chemoresistance and the EH/Ci/Mi regimen’s broad spectrum of anti-cancer cell capability

**Figure 4:**
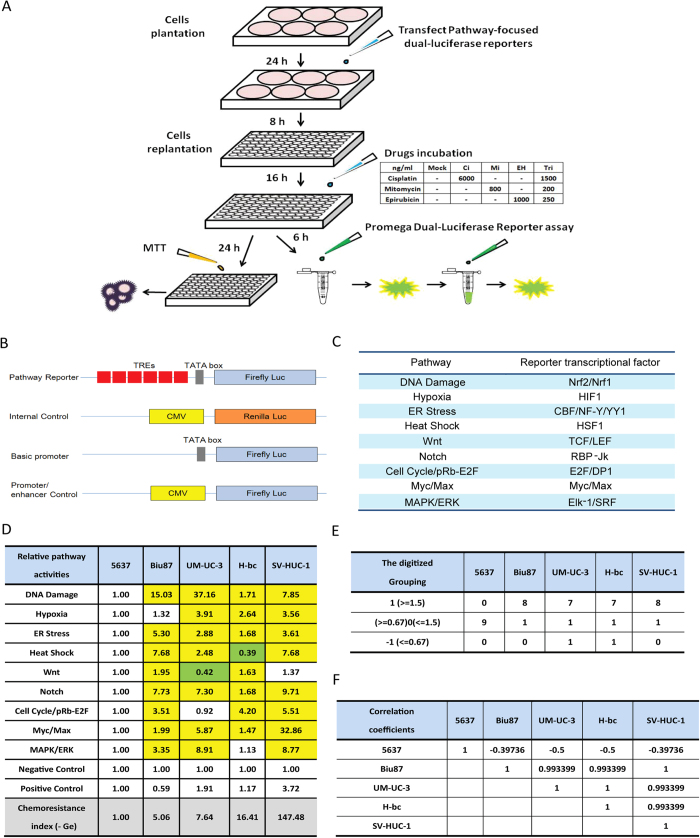
The activity of nine pathways in five cell lines of distinct chemoresistance. **(A)** The scheme of pathway analysis. (**B**) The firefly luciferase reporter constructs were used for the pathway analyses.1) “Basic promoter” reporter (the negative control in D), 2) “Pathway reporter”, where a repeat of the DNA motif bound by the master transcription factor in each pathway is inserted upstream of the firefly luciferase gene in “Basic promoter” reporter, 3) “Internal control”, the *Renilla* luciferase gene is under the control of both CMV viral promoter and enhancer (to control for transfection efficacy), and 4) “Promoter/enhancer control”, the firefly luciferase gene is under the control of both CMV viral promoter and enhancer (the positive control in D). **(C)** The nine pathways and their master transcription factor. (**D**) The normalized activity of each pathway versus the negative control in 5637 was set at 1 (the original data is Table S5-S9). The normalized pathway activity of all five cell lines was normalized to that in 5637 (fold) in Table. The pathways with activity higher by 0.5 fold or more are marked in yellow and lower by 0.5 fold in green. (**E)** The relative pathway activities were digitalized and sorted in each of the following three groups: (1), activated: the pathways with the activities elevated by no less than 0.5 fold by the drug; (−1), inactivated: the pathways with the activities down by no less than 0.5 fold by the drug, and (0), not responding: none of the above two cases. (**F)** The correlation coefficients of each pair of the indicated cell lines.

The signaling pathway(s) impacted by conventional chemotherapeutic agents remain enigmatic, despite years of research effort. To provide the signaling pathway based mechanisms for BCa chemoresistance, we used a Signal Pathway Finder reporter system (Qiagen) (Fig. 4A–C) to determine, in 5637 (chemo-sensitive), Biu87 (moderately resistant), UM-UC-3 and H-bc (resistant) BCa cell lines, and SV-HUC-1 cell lines, the activities of the following nine signaling pathways that are known to be involved with cell survival and death of both healthy and disease cells: DNA Damage response, Hypoxia, ER Stress, Heat Shock, Wnt, Notch, Cell Cycle/pRb-E2F, Myc/Max, and MAPK/ERK pathways and normalized them (Table S5-S9). The relative pathway activity between more resistant cell lines: Biu87 (Table S6), UM-UC-3 (Table S7), H-bc (Table S8), and SV-HUC-1 (Table S9) over the most chemo-sensitive cell line, 5637 (Table S5) was determined and analyzed (Fig. 4D). Eight pathways exhibited higher activities in Biu87 and SV-HUC-1 than in 5637 and 7 pathways showed higher activity in UM-UC-3 and H-bc cells than in 5637, by more than 0.5 fold (Fig. S2D). Therefore, a higher level of the basal activity of most pathways tested positively correlated with the chemoresistant state of BCa cells.

We then determined the correlation coefficient between the overall activity of these nine pathways and the chemoresistant state of these five cell lines. The pathway activity was digitized as follows: (1) an active state: the signaling pathway with an activity higher by 0.5 fold than in 5637; (−1) an inactive state: the signaling pathway with an activity lower by 0.5 fold than 5637; and (0) an inert state: the signaling pathway with an activity being neither of the above. The overall state of activity of these pathways in each cell line were summarized (Fig. 4E) and the correlation coefficient of each pair of cell lines was calculated (Fig. 4F) (see Supplementary material 4 the Pearson correlation analysis).

The correlation coefficients of the overall nine pathway activity between 5637 and four chemoresistant cell lines were negative (−0.397, −0.5, −0.5, and −0.397), and indicating the distinct state of 5637 from the other four cell lines, these figures in each pair were close to 0.99 (Fig. 4F). It was anticipated that the drug-triggered pathway activities also reflect the extent of chemoresistance of the cells. Cells were subjected to a 6 hrs treatment with either the EH/Ci/Mi regimen (EH,250 ng/mL, Ci, 1500 ng/mL and Mi, 200 ng/mL) or each component drugs at a fourfold dose: EH (1000 ng/mL), Ci (6000 ng/mL) and Mi (800 ng/mL) as well as two other drugs. The EH/Ci/Mi regimen’s ACS for BCa cells was significantly lower than that of each fourfold dosed component drugs (and the other two drugs), while it was higher than a fold dose of Ci for SV-HUC-1 cells than a fourfold dosed Ci (Table S4). The 6 hrs post-drug-triggered pathway activities were measured (Table S5-S9), normalized with the basal level activities (Table 4), digitalized (Table 5), and summarized (Table 6) for the correlation analysis of pathway activities by each single drug treatment and the EH/Ci/Mi regimen (Table 7). Taking 0.917 as the cutoff, the EH/Ci/Mi regimen’s effect on the pathway activity was the same as that of all the three component drugs in 5637 cells. On the contrary, only one component drug (Mi, in UM-UC-3, EH, in H-bc) or two drugs (Mi and EH, in Biu87) triggered pathway activity that was the same as the EH/Ci/Mi regimen’s. This observation suggests that the ability of the EH/Ci/Mi regimen to evoke the pathway response by one or more fourfold dosed single drugs may form the mechanistic basis for its a broad spectrum of cancer cell killing. Although the analysis of this level grouped 5637 and SV-HUC-1 (the most resistant) cell lines together, the detailed difference is obvious. The numbers of drug-activated pathways were from 5–8 in 5637 in comparison to 3–4 in SV-HUC-1 cells, while the numbers of inert pathways to drug treatment in 5637 were 1–3 versus 5–6 in SV-HUC-1 cells (Table 5 and Table 6).

### The EH/Ci/Mi regimen inhibited the *in vivo* growth of bladder cancer cell line derived tumor xenografts in nude mice more effectively than its mono-drug or bi-drug counterparts

**Figure 5:**
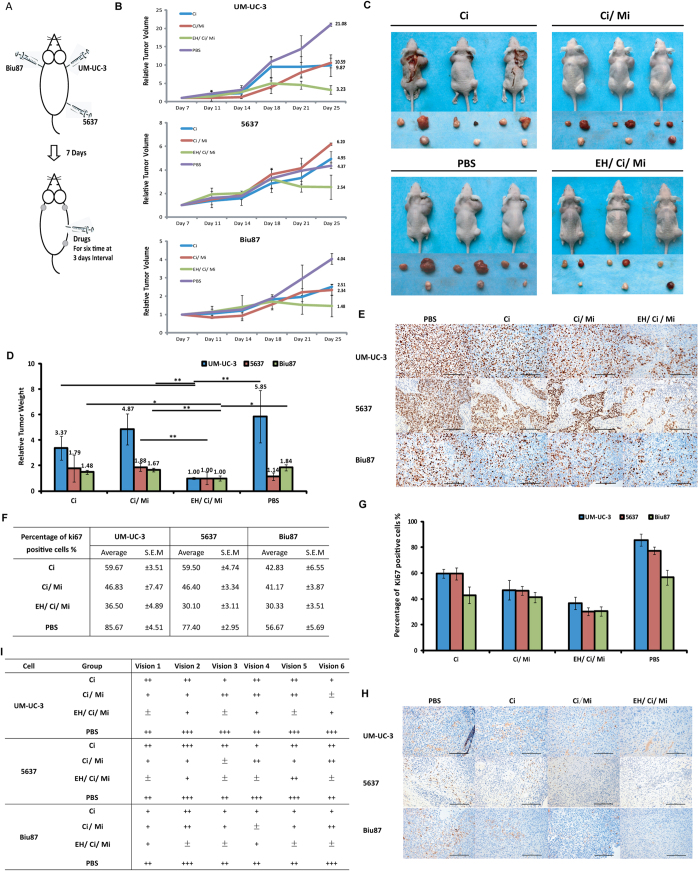
The effects of the EH/Ci/Mi regimen on*in vivo* tumor growth in a xenograft/nude mice model. **(A)** The experimental scheme. **(B)** Relative growth profile of UM-UC-3, 5637, and Biu87 tumor xenografts in nude mice. Y-axis: The mean and ± SD of the relative tumor volume (3 mice per group) measured on the indicated day over the volume on day 7. **(C)** The tumors and nude mice on day 28 when the experiment was terminated. **(D)** Tumor weights on the 28^th^ day. The mean and ± SD of the relative tumor weight (3 mice per group) over that the EH/Ci/Mi regimen was plotted *p < 0.05; **p < 0.02. **(E)** The representative Ki67 images of tumor xenografts subjected to immunological staining with Ki67 antibody. Photographs were taken under a microscope at 200 × magnification, scale bar units: 100 μm. **(F)** Summary of Ki67 positive cell counts (2 slides per tumor × 3 tumors in each group). Data were expressed as the mean ± S.E.M. and plotted (**G**). (**H**) The representative CD34 immunostaining images. Photographs were taken under a microscope at 200 × magnification.**(I)** Summary of CD34 positive cells for each group (2 slides per tumor × 3 tumors in each group). Immunohistochemistry staining level of CD34 positive vasculature were described as: ±, no staining; +, weak staining; + +, moderate staining; and + + +, strong staining.

We then compared the *in vivo* anticancer activity of the EH/Ci/Mi regimen with its mono-and bi-drug counterparts on the 5637, UM-UC-3, or Biu87 cells derived tumor xenografts in nude mice. Since there is no established conversion rule for the drug dose in cell culture to in nude mice, we used 75 μg per mouse for the mono-drug treatments and 25 μg per drug per mouse for both bi-drug and tri-drug combinations, based on the maximum tolerated drug dose recommended for these drugs^31^,^32^,^33^. To minimize the inter-mouse bias’s effect on tumor growth, three different BCa cell line-derived tumor xenografts were individually subcutaneously established on the back of the twelve mice (Fig.5A). A group of three mice were intraperitoneally injected with PBS (Phosphate balanced solution, the no-drug control), Ci, Ci/Mi, and the EH/Ci/Mi regimen on day 7 (after injection of the cancer cell lines), and at a three-day interval four more times. The tumor volumes were measured once every three days and were normalized by the volume on day 7. As shown in Fig. 5B–F, the increase in tumor volume on day 28 was 21 and 6.20 in PBS respectively for UM-UC-3 and Biu87, 10.58 and 4.95 in Ci, 8.87 and 4.37in Ci/Mi treated, 2.25 and 2.54 in the EH/Ci/Mi treatment mice (Fig. 5B). The relative tumor volume of 5637 cells in the EH/Ci/Mi group (2.54) was lower than that in Ci (6.2) and Ci/Mi groups (4.95)(Fig.5B). The conclusion of the better anti-cancer effect of the EH/Ci/Mi regimen than Ci and Ci/Mi regimens was further supported by the different tumor masses on day 28 when the animal study ended. Taking the tri-drug group as 1, relative tumor weights in the PBS group were 5.85, 1.14, and 1.84 for UM-UC-3, 5637, and Biu87 derived tumor, and in Ci and Ci/Mi groups were 3.37, 1.79, and 1.48 and 4.87, 1.88, and 1.67, for UM-UC-3, 5637, and Biu87 derived tumor, respectively (Fig. 5D). Elevated levels of both Ki67 and CD34 proteins indicate active cell proliferation^34^,^35^ and increased blood vasculature formation^36^ in the tumor mass. The percentage of Ki67-positive cells and the numbers of CD34 positive vascular structures in tumor xenografts were reduced by Ci, Ci/Mi, and EH/Ci/Mi treatments relative to PBS controls (Fig. 5F–I). The highest reduction was with the tri-drug combination and the lowest with the single drugs, which is consistent with the effects on tumor growth of this tri-drug combination.

## Discussion

Comprehensive cancer genomic studies in the last few years have repeatedly demonstrated that genetic heterogeneity in cancer cells differentiates not only in tumors from different individuals, but also different lesions, different part in a single lesion, or different cancer cells in a cancer patient^37^,^38^. This^39^ has been regarded as the major cause of failure in cancer treatment. Therefore, optimizing a multi-drug regimen capable of killing a broad spectrum of cancer cells with different single drug-resistant profiles should be done on a panel of established cancer cell lines to capture disease diversity in clinics. BCa consists of two major pathological subclasses, approximately 90% of cases in transitional cell carcinoma and 6% of cases in squamous cell carcinoma. In this study, seven TCC and one squamous carcinoma cell lines that dramatically vary in chemoresistance to five of six drug exposures (Fig. 2G) were used, with the intent to better reflect the heterogeneous spectrum of BCa. We used the FSC platform, DE algorithm guided experimental testing (Fig.1) to assess the cell killing capability of 100 out of 15,625 possible combinations of a set of six drugs at five doses in four rounds of testing. A tri-drug regimen (EH/Ci/Mi, 1500 ng/mL Ci, 200 ng/mL Mi, and 250 ng/mL EH) was able to kill 82.86% to 99.52% of cancer cells of 7 TCC and, 93.75% of 1 squamous BCa cell line in comparison with 52.53% of the immortalized normal cells (Table S4). The ideal state of the EH/Ci/M regimen was confirmed from a statistic modeling analysis of the ACS readouts of 100 combinations tested (Fig. S1). This tri-drug regimen killed more BCa cells at a 3 fold lower concentration than that of each component drugs (Table S4 and Fig. S3), indicating a synergistic interaction among these three drugs when used together. We also showed that the EH/Ci/Mi regimen was more potent than mono-drug and bi-drug combinations in inhibiting the growth of tumor xenografts derived from 5637, UM-UC-3, and Biu87 cell lines in nude mice (Fig. 5). As shown in Table S4, the tri-drug combination killed bladder cancer cells more efficiently than single components, and provided a kill range from 0.48% (H-bc) to 17.14% (EJ). Interestingly, the tri-drug combination dramatically killed a high rate of resistant cell H-bc, and thus may be able to cure some therapeutic resistant breast cancers. Since the EH/CI/Mi regimen was the most favorable with TW (41.15%) and TW index: (34.83%, and Table S4 and Fig. S3), it is expected to be able treat a broad spectrum of bladder cancer patients than any single drug.

The current anti-bladder cancer combination chemotherapies are chiefly cisplatin-based: CMV (cisplatin, methotrexate, and vinblastine), M-VAC (methotrexate, vinblastine, adriamycin, and cisplatin)^40^, and GC (gemcitabine plus cisplatin)^41^,^42^,^43^. Recent reports suggest a combined use of EH and Mi for BCa treatment^44^,^45^,^46^. EH’s mechanism of action is different from Ci and Mi^46^,^47^,^48^ that covalently bind DNA and repress both replication and transcription of DNA, leading to cell death^49^,^50^,^51^,^52^. As an intercalating agent, EH affects the secondary structure of DNA and therefore inhibits transcription and induces apoptosis^53^,^54^. Thus, use of these drugs together at a lower dose can simultaneously target different mechanisms for cancer cell killing along with a reduced level of cytotoxicity of the normal cells as described in this study.

To understand why the EH/Ci/Mi regimen is capable of killing a broad spectrum of BCa cells but each component drug at a three fold higher dose failed, we analyzed the basal and drug-triggered activities of nine cancer associated signaling pathways in four bladder cancer cell lines (5637, UM-UC-3, Biu87, and H-bc) and an immortalized non-transformed bladder epithelial cell line (SV-HUC-1) (Table. S4): DNA damage response, Hypoxia, ER Stress, Heat Shock, Wnt, Notch, Cell cycling, Myc/Max, and MAPK/ERK pathways (Table 4, 5, 6, 7 and Table S5-S9). Consistent with the chemoresistance of these cell lines, the basal level activities of these signaling pathways were collectively higher in chemoresistant cell lines (UM-UC-3, Biu87, H-bc, and SV-HUC-1) than the most sensitive cell line, 5637 (Fig. 4 and Table S5-S9). We also found that the subsets of pathways activated by the tri-drug combination were similar to at least the set of pathways triggered by the dominant drug in all cell lines tested (Table 4, 5, 6, 7 and Table. S5-S9). This observation offers a mechanistic explanation for why the EH/Ci/Mi regimen can effectively kill all the cancer cell lines tested, but the component drugs at a fourfold doses failed.

By identifying the EH/Ci/Mi regimen as a particular example for discovering effective BCa specific chemotherapeutic regimens, we demonstrated both robustness and general applicability of the FSC platform to develop effective drug (chemotherapeutics, biologicals, and target therapeutics) combination therapy for cancer.

## Methods

### Experimental Design

Each experiment was performed three times. All animals were adult BALB/C male nude mice 8–12 weeks of age and experiments were performed in accordance with the National Institutes of Health Guide for the Care and Use of Laboratory Animals and approved by the animal research committee of Harbin Medical University, China.

### Cell culture conditions and treatments

Cell lines (Table S1) used in this study were one normal uroepithelium cell line, SV-HUC-1^55^, six muscles invasive (EJ^56^, J82, UM-UC-3, T24, 5637, and H-bc) bladder cancer cell lines, one superficial transitional bladder cancer cell lines (Biu87) 57, and one squamous-cell carcinoma cell line (SCaBER). The clinical grade of the chemotherapeutics^58^,^59^ (NCI Dictionary of Cancer Terms, http://www.cancer.gov/dictionary) was provided by the First Affiliated Hospital of Harbin Medical University: Pirarubicin (Pi, Wanle, Shenzhen), Paclitaxel (Pa, Taiji, Sichuan), Adriamycin (Ad, Pfizer, Jiangsu), Epirubicin Hydrochloride (EH, Haizheng, Zhejiang), Hydroxycamptothecin (Hy, Lishizhen, Hubei), Cisplatin (Ci, Haosen, Jiangsu), Gemcitabine (Ge, Haosen, Jiangsu), and Mitomycin (Mi, Haizheng, Zhejiang). Drug induced cell death was determined by a thiazolyl blue tetrazolium blue (MTT, 490 nm reading) based cell proliferation assay in the cultured cells following treatment by various concentrations of drugs for 72 hours, as previously described^60^. The relative chemoresistance to each drug of all cell lines was calculated with the lowest IC_50_ as a reference. The overall chemoresistance of each cell line was described as the “chemoresistance index”, the numerator of the total relative IC_50_ to all the drugs over the number of drugs to rank the overall resistance state of each cell line to these six chemotherapeutics collectively.

### Optimization of the tri-drug regimen via the FSC platform in eight BCa cell lines

“Differential Evolution” algorithm guided experimental optimization (DE) consists for three iterative operations: generating drug combinations for experimental testing, acquiring the efficacy of the generated drug combinations from biological assays, and recalculating further improved drug combinations using a feedback search algorithm. We first tested twenty drug combinations at randomized doses. These drug combinations were generated from a random integer matrix generator, coded in MATLABTM language. These twenty drug combinations were then experimentally tested for cancer cell cytotoxicity. The experimental data were then subjected to calculation with a DE algorithm for new drug combinations based on three major mathematical calculations: “Mutation”, “Crossover” and “Selection”. The equations for each step can be found in the main text. The equations for each step are coded in MATLABTM language.

### Statistical Modeling on the relationships between cell survival and drug dose

Statistical analysis of experimentally measured cell survival rates was done for each individual cell line. The readouts (ACS) from 100 tested options for each cell line were pooled together and a polynomial regression model was built. The polynomial regression modeling is commonly used to mathematically describe the relationship between Y’s (in our case, the cell death) and X’s (in our case, the drug doses) using the R language environment. The polynomial regression model was computed by function “g = lm{stats}” and simplified by function “g = step(stats)” in R programming.

### The pathway analysis

The pathway analysis was performed with the Qiagen’s Cignal Finder Pathway Reporter Array systems according to the manufacturer’s instructions. The pathway-focused dual-luciferase reporter construct is a mixture of an inducible transcription factor responsive firefly luciferase reporter and constitutively expressing *Renilla* luciferase construct. Transcriptional response element (TRE) sequence response elements to each specific transcriptional factor triggered by the different signaling pathways are positioned before the TATA box of the firefly luciferase reporter gene. The extent of activity of the reporter reflects the level of activity of the particular signaling pathway. The CMV controlled *Renilla* luciferase expression vector is co-transfected into the cell as an internal control. A basic reporter construct that lacks the transcriptional factor binding sequence and could not be induced by any transcription factors is used as a negative control. Cells transfected with the CMV directed firefly luciferase reporter gene serve as a positive control. Briefly, cells seeded in 6-well plates were transfected with Cignal finder pathway arrays according to the manufacturer’s instructions (Qiagen)^61^. Then cells were seeded onto 96-well plates. Ci (6000 ng/mL), Mi (800 ng/mL), EH (1000 ng/mL), or the Ci/Mi/EH combination (Ci, 1500 ng/mL, Mi, 800 ng/mL and EH, 1000 ng/mL) were then added to the cells 24 h post-transfection. After 6 h of incubation with the different drugs at 37 ºC, cells were lyzed and a dual luciferase assay (for firefly and *Renilla* luciferase) was performed using a Promega GloMax 20/20 illuminometer. The firefly luciferase activities were normalized over the *Renilla* luciferase activities after background activity determined by luciferase activities of negative controls was subtracted. A triple test of cell survival under these drug doses was also performed by MTT assay following 18 h incubation at 37 ºC.

### Pearson correlation analysis of the signaling pathway data in chemoresistant distinct cell lines

The levels of pathway activities were digitized as follows: activated (1): if pathway activity was higher than 1.5-fold over the activity in 5637 cells (Table 5) or than the no drug control; repressed (−1), if the activity was 0.5- fold lower than in 5637 cells (Table 6) or than the no drug control, and null (0) if neither of the above. Correlation coefficients between two cell lines or between drug treated cells and untreated cells were calculated (Table 7). The definition and calculation equations for correlation coefficients can be found in the main text. To calculate the correlation coefficients between the two cell lines we used R language statistics. Function “cor{stats}” was used to generate the correlation matrix between multiple cell lines.

### The *in vivo* study

Cells (1 × 10^6–7th^/100 μL) embedded in BD Matrigel™ Matrix^62^ were subcutaneously injected at three sites in the back of each mouse (3–4 mice per treatment). On days 7–12 following cell injection, the animals were treated with the different drugs by an intraperitoneal injection, which was repeated every three days. Tumor volumes were monitored/calculated using the equation V = W^2^L × 0.5 (where W and L represent the largest and second largest tumor diameters (mm)) and then plotted. The tumor mass were weighed, and fixed/sliced for immunohistochemistry after the animals were sacrificed.

### Immunohistochemical analysis

Immunostaining was performed on 5 μm slices of formalin fixed paraffin-embedded tumor xenografts using antibodies from Gene Tech Company (Shanghai, China) against both Ki67 and CD34. Pictures were taken by a LEICA DM 4000B microscope and the relative level of each protein was calculated using LEICA software. The relative staining per antibody (in percentage) in the mock treated over chemotherapeutic treated tumors was calculated and plotted.

### Statistical Analysis

Two sets of tumor xenograft/nude mice studies were carried out. The *in vivo* study involved four groups with three mice per group. Both mean ± S.D of tumor volume and weight were calculated. Student’s t-test was used for statistical comparisons. P values less than 0.05 were considered statistically significant.

## Additional Information

**How to cite this article**: Liu, Q. *et al.* Preclinical optimization of a broad-spectrum anti-bladder cancer tri-drug regimen *via* the Feedback System Control (FSC) platform. *Sci. Rep.* **5**, 11464; doi: 10.1038/srep11464 (2015).

## Supplementary Material

Supplementary Information

## Figures and Tables

**Table 1:** The 1^st^ round of iteration.

**Combinations**		**1**	**2**	**3**	**4**	**5**	**6**	**7**	**8**	**9**	**10**	**11**	**12**	**13**	**14**	**15**	**16**	**17**	**18**	**19**	**20**
Drug concentration (ng /ml)	Pi	1.6	8	8	1.6	8	0.32	40	0.32	0.32	8	1.6	8	40	40	0.32	40	40	8	1.6	8
	Pa	32	6.4	32	1.28	32	160	1.28	6.4	6.4	160	1.28	160	1.28	1.28	1.28	1.28	32	6.4	6.4	1.28
	EH	10	2	50	10	50	10	50	250	10	10	50	50	250	250	10	50	50	50	250	2
	Ci	300	300	300	12	60	1500	60	1500	300	12	60	1500	300	60	1500	12	1500	1500	1500	300
	Ge	5	1	0.04	5	5	0.2	0.2	1	0.2	0.2	0.04	5	5	0.04	1	1	5	5	0.2	0.04
	Mi	8	8	8	8	40	1000	200	1000	1000	200	8	40	40	8	40	200	200	8	200	40
Cell survival (%)	5637	24.58	43.82	45.43	65.40	42.03	1.23	26.47	0.51	11.57	16.17	62.93	13.59	26.19	47.21	30.10	25.27	6.62	23.94	1.99	48.26
	H-bc	52.57	56.01	52.30	59.60	58.01	18.96	48.40	3.40	42.60	51.76	57.78	26.84	30.01	33.02	33.64	44.03	15.80	29.12	5.48	58.13
	J-82	43.69	55.27	50.62	72.9	46.08	6.07	23.75	6.62	11.53	26.82	65.16	13.4	18.67	31	24.3	24.77	7.63	16.23	8.26	56.8
	T24	36.41	44.71	45.20	62.06	39.05	7.97	32.91	11.67	20.07	29.74	61.60	14.80	17.25	24.05	22.03	34.08	11.21	15.78	8.73	39.38
	EJ	46.55	51.31	40.41	69.1	41.61	30.67	37.67	21.79	35.01	36.4	56.02	36.62	22.28	28.35	45.2	34.79	31.27	37.86	23.63	53.67
	Biu87	21.67	49.15	51.1	26.78	31.5	7.64	48.37	16.09	20.32	37.65	72.26	8.56	17.59	33.22	14.02	45.38	12.87	14.05	13.3	65.93
	ACS (%)	37.58	50.04	47.51	59.31	43.05	12.09	36.26	10.01	23.52	33.09	62.63	18.97	22.00	32.81	28.21	34.72	14.23	22.83	10.23	53.70

The combinations (1–20) varied with the dose (ng/mL) of six drugs and had different effects on cell survival (%). The ACS (%) summarized from the cell survival of each cell line under the treatment of each combination.

**Table 2:** The 4^th^ round of iteration.

**Combinations**		**1**	**2**	**3**	**4**	**5**	**6**	**7**	**8**	**9**	**10**	**11**	**12**	**13**	**14**	**15**	**16**	**17**	**18**	**19**	**20**
Drug concentration(ng /ml)	Pi	40	8	8	40	0	0	1.6	0	0	40	1.6	1.6	1.6	1.6	1.6	8	8	0.32	8	8
	Pa	160	6.4	0	1.28	160	1.28	0	0	0	160	160	0	6.4	0	0	6.4	6.4	32	160	0
	EH	250	250	50	50	250	50	50	50	250	0	2	2	2	2	50	2	50	50	0	0
	Ci	1500	12	60	300	1500	60	1500	60	1500	1500	300	1500	1500	0	0	60	60	300	1500	300
	Ge	0	0	1	0.2	0	0	5	5	0.2	5	0.04	1	1	0.2	5	0.2	5	0.2	5	0.2
	Mi	1000	200	200	1000	1000	8	8	8	8	0	8	0	40	200	8	1000	200	0	0	1000
Cell survival(%)	Biu87	1.06	13.15	10.83	3.44	1.76	67.33	2.49	19.33	4.74	2.33	19.08	3.03	3.98	11.15	16.07	4.11	4.84	32.59	2.01	2.97
	J-82	1.09	16.03	24.08	3.20	1.55	81.67	10.85	83.10	11.47	9.01	46.99	17.53	13.42	22.31	83.37	5.41	15.91	55.90	11.47	3.26
	EJ	10.47	33.23	44.31	20.32	13.79	95.23	49.72	89.61	48.27	39.92	65.19	59.73	51.97	42.01	86.61	23.16	37.99	73.87	49.02	18.98
	ACCS (%)	4.21	20.80	26.41	8.99	5.70	81.41	21.02	64.01	21.49	17.09	43.75	26.76	23.12	25.16	62.02	10.89	19.58	54.12	20.83	8.40
	5637	12.67	—	—	49.82	31.48	—	58.46	—	54.30	54.91	—	—	—	—	—	58.89	63.70	—	59.90	58.19
	T24	6.71	—	—	6.07	6.53	—	67.09	—	60.35	64.83	—	—	—	—	—	14.88	32.74	—	62.31	11.91
	UM-UC-3	4.66	—	—	19.97	11.74	—	39.16	—	35.24	25.80	—	—	—	—	—	19.74	31.35	—	34.55	44.89
	H-bc	0.08	—	—	69.34	1.83	—	79.74	—	73.68	72.95	—	—	—	—	—	76.52	77.57	—	69.74	72.60
	ACCS (%)	4.59	—	—	21.52	8.58	—	38.44	—	36.01	33.72	—	—	—	—	—	25.34	33.01	—	36.13	26.60
Combinations		21	22	23	24	25	26	27	28	29	30	31	32	33	34	35	36	37	38	39	40
Drug concentration(ng /ml)	Pi	0	40	8	40	40	0	0	40	40	40	0	0	0	8	8	1.6	40	0	8	0.32
	Pa	32	0	160	0	0	32	0	0	160	32	160	6.4	0	0	0	160	0	32	0	0
	EH	2	50	0	250	250	50	10	50	50	0	10	0	10	50	0	2	50	250	50	250
	Ci	300	12	0	300	60	0	1500	0	300	12	1500	1500	0	1500	1500	0	1500	1500	1500	1500
	Ge	5	0	0.2	5	0	5	1	1	5	0	0.2	1	5	0.2	5	0.2	5	0.2	5	0
	Mi	0	200	200	40	0	1000	0	200	200	1000	0	1000	1000	1000	40	1000	1000	1000	40	200
Cell survival(%)	Biu87	11.37	13.41	13.91	5.62	19.78	2.76	4.77	12.09	5.70	6.88	2.83	1.70	2.42	1.41	2.23	3.39	1.73	2.01	3.74	2.17
	J-82	69.36	21.93	24.21	25.25	65.75	8.46	20.82	28.68	19.46	9.93	15.54	2.21	9.93	1.86	12.82	8.68	1.71	1.61	12.36	7.14
	EJ	77.62	36.29	36.91	44.56	66.58	25.51	63.19	37.58	39.91	20.74	40.61	15.55	26.85	16.89	47.02	21.00	13.66	15.55	48.61	28.13
	ACCS (%)	52.78	23.88	25.01	25.14	50.70	12.25	29.60	26.11	21.69	12.51	19.66	6.49	13.06	6.72	20.69	11.02	5.70	6.39	21.57	12.48
	5637	—	—	—	—	—	55.04	—	—	—	55.21	56.03	41.74	67.91	38.98	72.15	53.50	47.58	48.65	74.74	39.77
	T24	—	—	—	—	—	8.76	—	—	—	10.50	63.41	4.91	8.76	6.51	56.17	8.21	5.96	5.54	58.66	5.62
	UM-UC-3	—	—	—	—	—	11.12	—	—	—	22.07	36.00	20.91	12.29	27.58	22.86	18.08	25.76	21.01	25.44	21.67
	H-bc	—	—	—	—	—	72.55	—	—	—	73.72	73.03	35.05	81.70	36.08	78.21	75.08	15.01	3.23	76.61	4.30
	ACS (%)	—	—	—	—	—	23.03	—	—	—	24.88	35.93	15.26	26.23	16.16	36.43	23.49	13.93	12.20	37.52	13.60

The combinations (1-40) varied with the dose (ng/mL) of six drugs and had different effects on the cell survival (%). The ACS (%) summarized from the cell survival of each cell line under the treatment for combination.

**Table 3:** 

**Combinations**		**1**	**2**	**3**	**4**	**5**	**6**	**7**	**8**	**9**	**10**	**11**	**12**	**13**	**14**	**15**	**16**	**17**
Drug concentration (ng /ml)	Pi	0	0	0	40	0	0	0	8	0	0	0	0	40	0	0	0	0
	Pa	0	0	160	0	0	0	32	0	0	0	0	160	0	0	0	0	160
	EH	0	250	0	0	0	50	0	0	0	0	250	0	0	0	0	250	0
	Ci	1500	1500	1500	1500	1500	1500	1500	1500	1500	1500	1500	1500	1500	1500	300	300	300
	Ge	0	0	0	0	5	0	0	0	1	0	0	0	0	5	0	0	0
	Mi	1000	1000	1000	1000	1000	1000	1000	1000	1000	200	200	200	200	200	1000	1000	1000
Cell survival(%)	Biu87	4.5	2	1.3	3.5	5.7	4.3	1.9	1.7	3.5	5.3	0.6	1.9	2.8	3.7	13.4	9.9	10.7
	EJ	17	15.4	10.4	16.7	20	22.4	14.3	20	19.4	27.7	16.7	25.1	22.5	25.7	30.9	29.8	23.5
	H-bc	29.5	4.79	19.82	5.84	7.57	5.11	42.68	8.5	29.95	41.03	12.43	13.56	17.24	32.23	57.1	29.7	44
	T24	3.6	1.6	3.7	1.6	4.8	3.2	3.9	3.8	4.5	5.6	6.5	1.6	3.5	8.9	7.09	29.5	7.69
	UM-UC-3	3	2.9	3	3.6	3.5	2.7	3	3.6	3.1	4.6	3.6	4.2	4.1	4.1	32	14.1	19.4
	ACS (%)	11.52	5.34	7.64	6.25	8.31	7.54	13.16	7.52	12.09	16.85	7.97	9.27	10.03	14.93	28.10	22.60	21.06
Combinations		18	19	20	21	22	23	24	25	26	27	28	29	30	31	32	33	34
Drug concentration(ng /ml)	140	0	0	0	0	40	0	40	40	40	8	0	0	0	8	0	0	40
	0	0	0	0	160	0	0	32	0	0	0	32	0	32	0	0	0	32
	0	0	0	250	0	0	0	0	50	0	0	0	50	250	250	250	250	50
	300	300	300	300	300	300	300	300	300	300	300	300	300	300	300	300	300	1500
	0	5	0	0	0	0	5	0	0	1	5	5	5	0	0	1	5	5
	1000	1000	200	200	200	200	200	200	200	200	200	200	200	200	200	200	200	200
Cell survival(%)	Biu87	13.3	6.8	36.4	21.7	27.2	30.7	8.4	30.4	29.3	24.2	14.9	12.5	14.5	28.1	27.3	22.7	13.2
	EJ	31.8	27	52.8	40.9	38.7	55.9	39.3	38.1	47.8	48.4	46.5	37.5	44.9	40.5	45.1	49.9	40.4
	H-bc	49.9	50.3	73.2	48.4	55.9	49.9	64.2	55	58.3	70.9	63	63.1	58.6	47.8	33.1	45.4	51.9
	T24	12.76	71.7	8.87	45.97	55.81	46.27	50.98	40.97	34.88	46.03	51.52	47.05	53.67	33.09	31.25	39.53	33.27
	UM-UC-3	25.5	34.6	64.5	30.1	32.3	22.1	49.6	37.1	30.9	43.4	58.7	41.1	49.7	19.7	17.1	22.4	21.2
	ACS (%)	26.65	38.08	47.15	37.41	41.98	40.97	42.50	40.31	40.24	46.59	46.92	40.25	44.27	33.84	30.77	35.99	31.99

Table 3. Further experimental testing of the EH/Ci/Mi regimen (1). The combinations (1–34) varied with the dose (ng/mL) of three drugs and the cell survival (%) of each treated cell line was measured for each of the ACS (%).

**Table 4:**
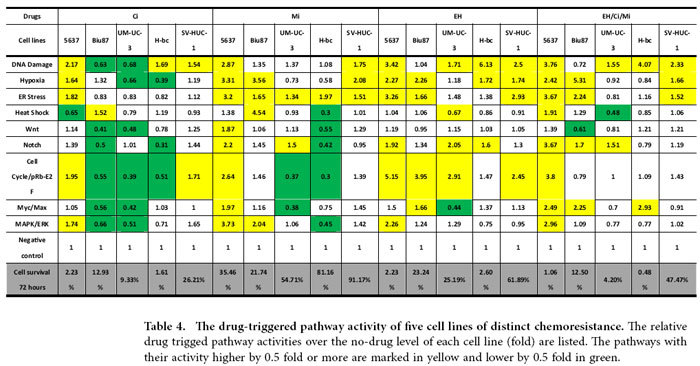
The drug-triggered pathway activity of five cell lines of distinct chemoresistance.

**Table 5:**
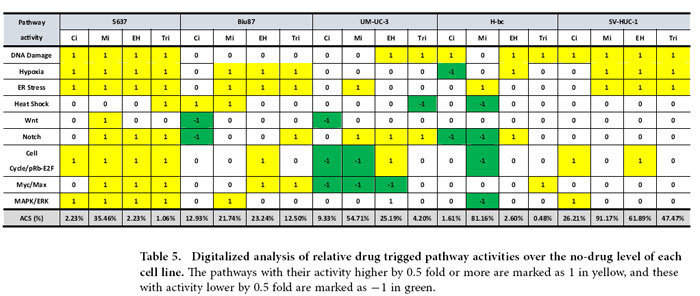
Digitalized analysis of relative drug trigged pathway activities over the no-drug level of each cell line.

**Table 6:**
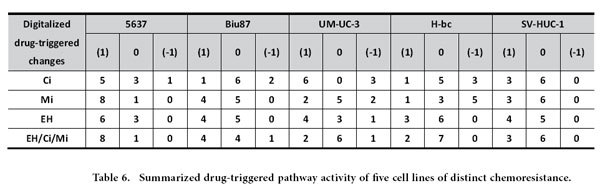
Summarized drug-triggered pathway activity of five cell lines of distinct chemoresistance.

**Table 7:**
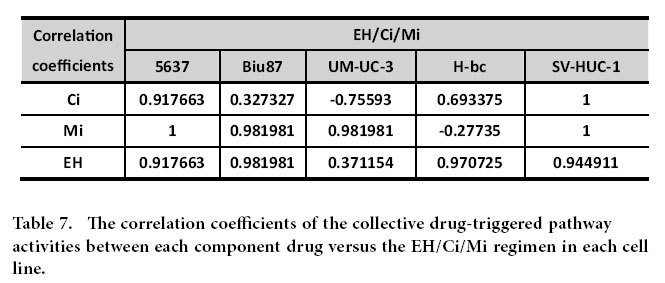
The correlation coefficients of the collective drug-triggered pathway activities between each component drug versus the EH/Ci/Mi regimen in each cell line.
