# Supplementary material for: Preclinical optimization of a broad-spectrum anti-bladder cancer tri-drug regimen via the Feedback System Control (FSC) platform
Source: Sci Rep. 2015 Jun 19;5:11464. doi: 10.1038/srep11464 (PMC5155572; doi:10.1038/srep11464)
Supplement: Supplementary Information [file srep11464-s1.doc]

**Title page of supplementary files**

**Preclinical optimization of a broad-spectrum anti-bladder cancer tri-drug regimen *via* the Feedback System Control (FSC) platform**

Qi Liu^1^†, Cheng Zhang^2^†, Xianting Ding^3^†, Hui Deng†^4^,Daming Zhang^5^, Wei Cui^1^, Hongwei Xu^6^, Yingwei Wang^2^, Wanhai Xu^7^, Lei Lv^4^, Hongyu Zhang^8^, Yinghua He^8^, Qiong Wu^1^, Moshe Szyf^9*^, Chih-Ming Ho^10*^, Jingde Zhu^4,8*^

**Supplementary files list:**

Supplementary Materials 1. Supplementary figures and tables.

Supplementary Materials 2. Differential Evolution search algorithm used in this study.

Supplementary Materials 3. Analysis of statistical modeling of four cell lines to decide component drugs for tri-drug combination.

Supplementary Materials 4. Use of Feedback System Control (FSC) Platform in the Present Study.

**Supplementary Materials 1. Supplementary figures and tables.**

**
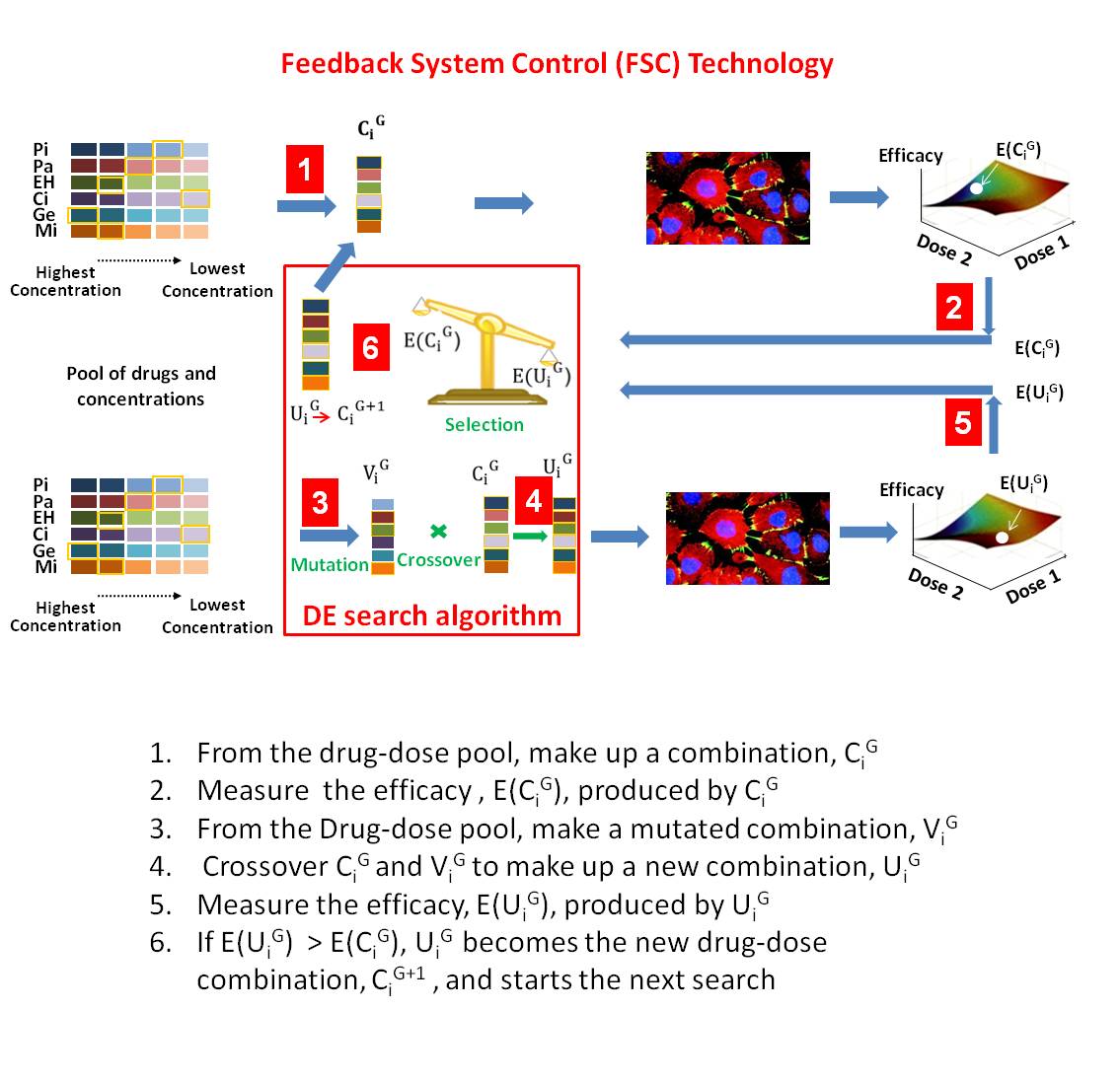
Fig. S1. Step by step illustration of the differential evolution algorithm used in this FSC experiment.** Four components are involved: 1. from the drug-dose pool from the IC_50_ profiling of BCa cell lines, make up a combination, C_i_^G^2. measure the efficacy, E(C_i_^G^), produced by C_i_^G^3. from the Drug-dose pool, make a mutated combination, V_i_^G^4. crossover C_i_^G^ and V_i_^G^ to make up a new combination, U_i_^G^5. measure the efficacy, E(U_i_^G^), produced by U_i_^G^6. if E (U_i_^G^) > E(C_i_^G^), U_i_^G^ becomes the new drug-dose combination, C_i_^G+1^ ,and starts the next search.


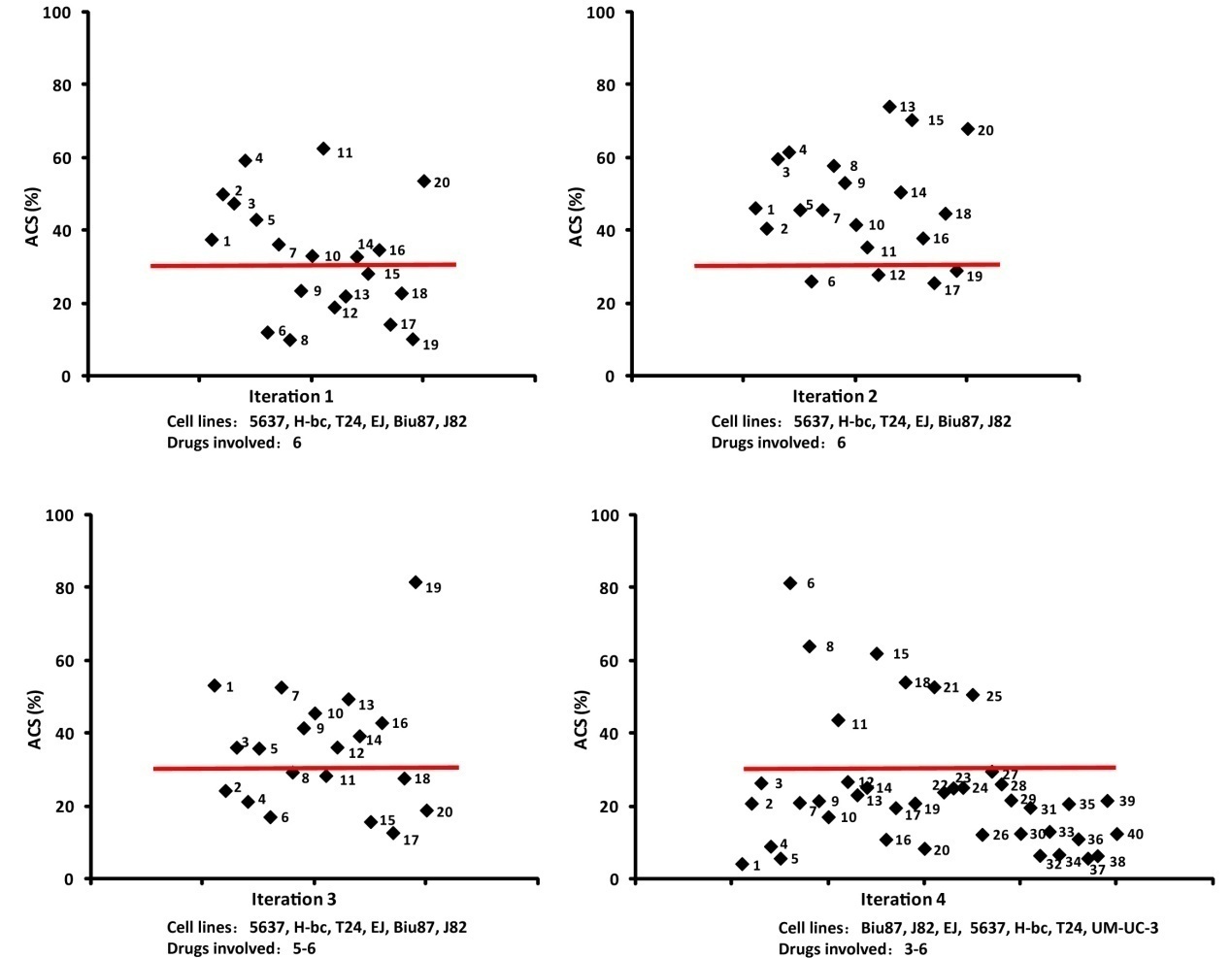


**Fig. S2. The number of effective combinations of the 1^st^ to 4^th^ rounds of iterations.** Y-axis, the ACS (%). The red line (30%) separates the effective combinations (<30%) from the ineffective (> or = 30%).


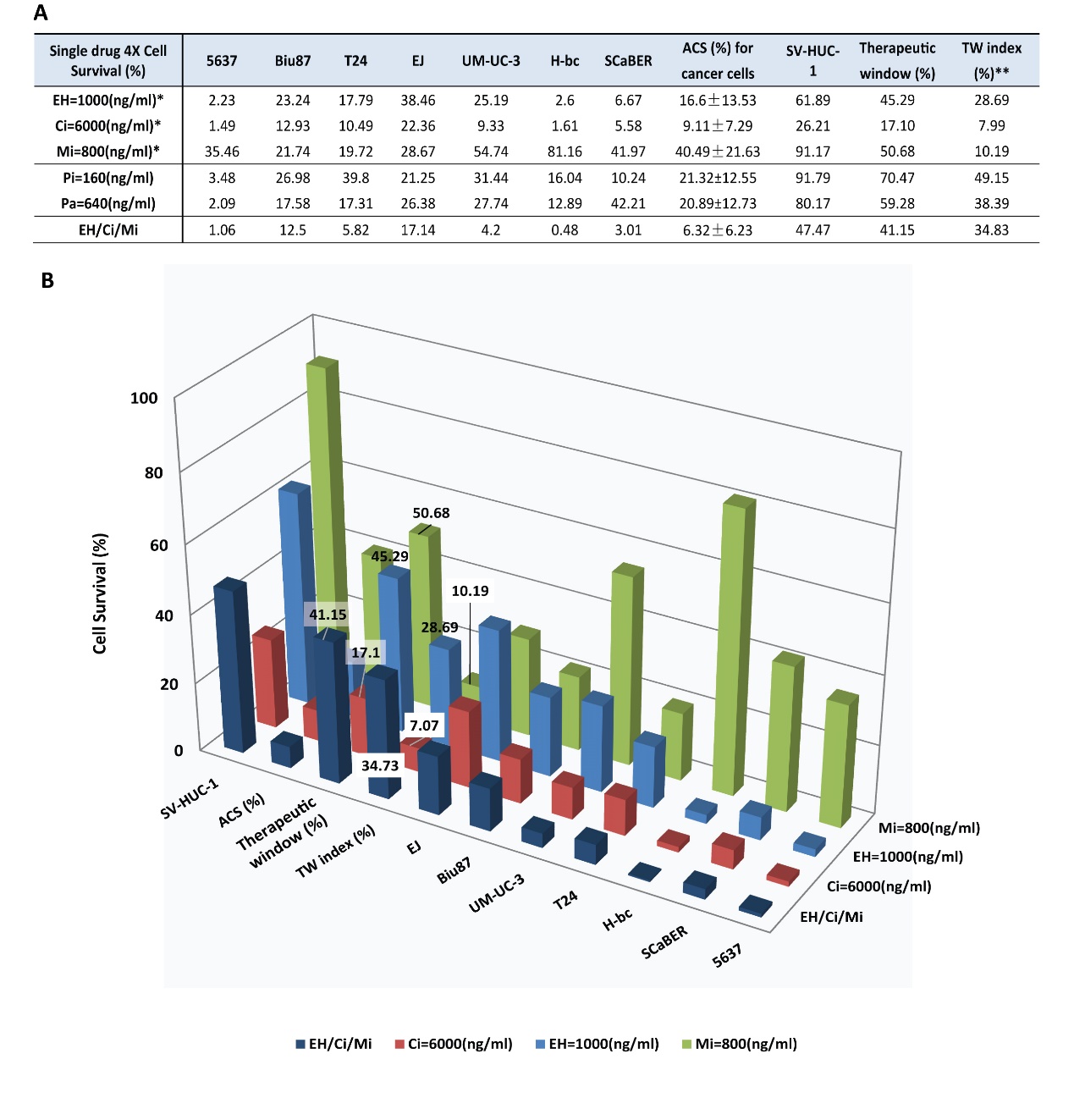


**Fig. S3. Histogram of further experimental testing of the EH/Ci/Mi regimen.** The EH/Ci/Mi regimen and five single drugs at a fourfold dose (ng/mL) and the cell survival (%) of each treated cell lines (**A**) were measured. Therapeutic window (TW) refers to the difference (%) in the treatment trigger cell survival between SV-HUC-1 and BCa cell lines. The therapeutic window index (TW index), referring to the difference (%) between the therapeutic window and the ACS % ranking of the EH/CI/Mi regimen and three component drugs at a threefold higher dose, were also calculated in (**A**). *, the drug dose was three fold higher than the dose of the same drug in the EH/Ci/Mi combination. **, the therapeutic index= Therapeutic window (%) - ACS% of single drug or EH/CI/Mi regimen. Histogram of ACS, TW, and TW index of the EH/Ci/Mi regimen and each of the three component drugs at a fourfold dose are shown in **B**.

| Cell line | ATCC | Medium ＋ 10% fetal serum (Invitrogen, USA) | Description |
| --- | --- | --- | --- |
| SV-HUC-1 | CRL-9520™ | Ham's F12 K(*5*) | Normal uroepithelium cell line |
| SCaBER | HTB-3™ | MEM | Squamous-cell carcinoma cell line |
| J82 | HTB-1™ | MEM | Muscle invasive transitional cell carcinoma line |
| UM-UC-3 | CRL-1749™ | MEM | Muscle invasive transitional cell carcinoma line |
| T24 | HTB-4™ | RPMI 1640 | Muscle invasive transitional cell carcinoma line |
| 5637 | HTB-9™ | RPMI 1640 | Muscle invasive transitional cell carcinoma line |
| EJ | Null | RPMI 1640 | Muscle invasive transitional cell carcinoma line, established by Marshall CJ in 1977 |
| H-bc | Null | RPMI 1640 | Muscle invasive transitional cell carcinoma line, established by cancer research Institute of Kunming Medical College, 1986 |
| Biu87 | Null | RPMI 1640 | Superficial bladder cancer, established by the Department of Urology of Beijing Medical University in 1987 |

**Table S1. Origin of cells and chemotherapy drugs**

| **Combinations** | | **1** | **2** | **3** | **4** | **5** | **6** | **7** | **8** | **9** | **10** | **11** | **12** | **13** | **14** | **15** | **16** | **17** | **18** | **19** | **20** |
| --- | --- | --- | --- | --- | --- | --- | --- | --- | --- | --- | --- | --- | --- | --- | --- | --- | --- | --- | --- | --- | --- |
| **Drug**  **concentration**  **(ng /ml)** | **Pi** | 8 | 0.32 | 40 | 0.32 | 8 | 8 | 1.6 | 8 | 40 | 0.32 | 1.6 | 0.32 | 1.6 | 0.32 | 8 | 40 | 1.6 | 40 | 40 | 1.6 |
|  | **Pa** | 6.4 | 1.28 | 6.4 | 32 | 1.28 | 6.4 | 32 | 160 | 1.28 | 160 | 1.28 | 32 | 1.28 | 6.4 | 6.4 | 160 | 160 | 160 | 32 | 32 |
|  | **EH** | 250 | 10 | 10 | 50 | 2 | 50 | 10 | 250 | 250 | 2 | 10 | 50 | 50 | 250 | 10 | 50 | 250 | 2 | 2 | 2 |
|  | **Ci** | 12 | 1500 | 12 | 60 | 300 | 1500 | 1500 | 60 | 300 | 60 | 12 | 12 | 60 | 1500 | 300 | 300 | 300 | 1500 | 60 | 12 |
|  | **Ge** | 0.04 | 0.04 | 1 | 0.04 | 5 | 1 | 5 | 1 | 0.2 | 1 | 0.2 | 5 | 5 | 1 | 0.04 | 5 | 0.2 | 0.2 | 0.04 | 0.2 |
|  | **Mi** | 200 | 200 | 40 | 40 | 200 | 1000 | 40 | 8 | 40 | 200 | 1000 | 1000 | 8 | 8 | 8 | 200 | 1000 | 40 | 1000 | 8 |
| **Cell**  **survival**  **(%)** | **5637** | 41.90 | 33.17 | 65.54 | 54.93 | 34.82 | 16.36 | 38.54 | 51.28 | 52.93 | 36.99 | 31.49 | 15.81 | 65.63 | 52.51 | 71.42 | 32.88 | 21.63 | 45.97 | 19.01 | 64.95 |
|  | **H-bc** | 76.86 | 75.17 | 73.66 | 75.43 | 75.43 | 67.23 | 73.62 | 78.67 | 76.38 | 74.87 | 74.70 | 65.85 | 80.87 | 74.35 | 78.20 | 67.23 | 63.73 | 69.78 | 66.02 | 66.49 |
|  | **Biu87** | 27.18 | 24.43 | 56.05 | 58.58 | 36.57 | 6.60 | 36.47 | 59.51 | 44.11 | 29.61 | 12.43 | 10.97 | 71.59 | 44.66 | 65.15 | 25.21 | 7.61 | 32.88 | 8.19 | 56.60 |
|  | **EJ** | 35.64 | 39.25 | 41.01 | 53.31 | 42.50 | 31.07 | 39.52 | 52.48 | 37.93 | 28.92 | 46.66 | 43.61 | 77.21 | 40.11 | 67.82 | 35.57 | 20.30 | 38.55 | 33.46 | 82.27 |
|  | **J-82** | 44.07 | 36.94 | 54.84 | 73.33 | 40.23 | 20.95 | 45.53 | 50.92 | 55.05 | 40.77 | 25.04 | 15.36 | 73.49 | 45.41 | 70.57 | 31.63 | 24.00 | 40.32 | 23.62 | 86.56 |
|  | **T24** | 51.61 | 34.64 | 67.07 | 53.90 | 44.68 | 14.28 | 40.47 | 54.26 | 52.51 | 38.60 | 22.32 | 15.67 | 75.85 | 46.27 | 69.44 | 34.97 | 16.44 | 40.72 | 23.79 | 51.16 |
|  | **ACS (%)** | 46.21 | 40.60 | 59.70 | 61.58 | 45.71 | 26.08 | 45.69 | 57.85 | 53.15 | 41.63 | 35.44 | 27.88 | 74.11 | 50.55 | 70.43 | 37.92 | 25.62 | 44.70 | 29.01 | 68.01 |

**Table S2. The 2^nd^ round of iteration**. The combinations (1-20) varied with the dose (ng/mL) of six drugs and had different effects on cell survival (%). The ACS (%) provides an overall measure of survival of all the cell lines in response to each combination.

| **Combinations** | | **1** | **2** | **3** | **4** | **5** | **6** | **7** | **8** | **9** | **10** | **11** | **12** | **13** | **14** | **15** | **16** | **17** | **18** | **19** | **20** |
| --- | --- | --- | --- | --- | --- | --- | --- | --- | --- | --- | --- | --- | --- | --- | --- | --- | --- | --- | --- | --- | --- |
| **Drug**  **concentration**  **(ng /ml)** | **Pi** | 1.6 | 0.32 | 40 | 0.32 | 0.32 | 8 | 0.32 | 8 | 0.32 | 40 | 1.6 | 0.32 | 0.32 | 40 | 40 | 40 | 0.32 | 8 | 1.6 | 1.6 |
|  | **Pa** | 6.4 | 160 | 32 | 6.4 | 6.4 | 6.4 | 6.4 | 6.4 | 1.28 | 1.28 | 160 | 160 | 160 | 160 | 6.4 | 6.4 | 32 | 1.28 | 6.4 | 6.4 |
|  | **EH** | 10 | 10 | 50 | 2 | 10 | 50 | 10 | 0 | 250 | 250 | 10 | 50 | 50 | 50 | 50 | 10 | 250 | 50 | 50 | 250 |
|  | **Ci** | 300 | 1500 | 60 | 1500 | 0 | 1500 | 12 | 1500 | 300 | 60 | 12 | 60 | 0 | 12 | 1500 | 12 | 1500 | 1500 | 60 | 1500 |
|  | **Ge** | 0.2 | 0.2 | 1 | 1 | 5 | 0.2 | 0.2 | 5 | 5 | 0.04 | 0.2 | 5 | 5 | 1 | 5 | 5 | 0.2 | 5 | 0.04 | 0.04 |
|  | **Mi** | 8 | 40 | 200 | 1000 | 1000 | 1000 | 200 | 40 | 40 | 8 | 1000 | 200 | 40 | 200 | 1000 | 200 | 1000 | 40 | 8 | 200 |
| **Cell**  **survival**  **(%)** | **Biu87** | 29.50 | 4.79 | 19.82 | 5.84 | 7.57 | 4.11 | 42.68 | 8.50 | 29.95 | 41.03 | 13.56 | 12.43 | 17.24 | 32.23 | 7.09 | 29.50 | 7.69 | 12.76 | 71.70 | 8.87 |
|  | **EJ** | 64.82 | 38.64 | 38.32 | 43.59 | 59.47 | 36.31 | 53.72 | 51.39 | 52.79 | 65.94 | 43.91 | 48.97 | 78.33 | 48.25 | 34.98 | 51.59 | 30.56 | 50.82 | 92.92 | 29.27 |
|  | **J-82** | 53.51 | 1.41 | 9.69 | 0.05 | 6.01 | 0.19 | 22.28 | 3.47 | 19.35 | 28.05 | 2.99 | 18.32 | 36.14 | 8.37 | 0.05 | 17.42 | 0.52 | 3.23 | 69.57 | 1.08 |
|  | **T24** | 49.79 | 9.55 | 25.80 | 8.27 | 14.12 | 7.94 | 55.31 | 7.90 | 17.90 | 21.93 | 15.88 | 20.25 | 31.85 | 31.98 | 8.60 | 26.34 | 10.41 | 9.75 | 86.21 | 9.84 |
|  | **5637** | 70.21 | 80.31 | 65.63 | 61.15 | 59.62 | 47.02 | 68.73 | 82.41 | 69.40 | 71.11 | 50.83 | 54.02 | 61.87 | 57.74 | 41.50 | 63.21 | 26.51 | 74.52 | 75.37 | 62.85 |
|  | **H-bc** | 51.65 | 10.95 | 57.71 | 8.79 | 68.66 | 6.80 | 73.38 | 22.34 | 59.52 | 45.37 | 43.16 | 63.29 | 71.34 | 57.27 | 2.25 | 69.48 | 0.52 | 15.02 | 94.07 | 1.52 |
|  | **ACS (%)** | 53.25 | 24.28 | 36.16 | 21.28 | 35.91 | 17.06 | 52.68 | 29.33 | 41.49 | 45.57 | 28.39 | 36.21 | 49.46 | 39.31 | 15.75 | 42.92 | 12.70 | 27.68 | 81.64 | 18.91 |

**Table S3. The 3^rd^ round of iteration**. The combinations (1-20) varied with the dose (ng/mL) of six drugs and had different effects on cell survival (%). The ACS (%) provides an overall measure of survival of all the cell lines in response to each combination.

| Combinations | | 1 | 2 | 3 | 4 | Pi | Pa | EH | Ci | Mi |
| --- | --- | --- | --- | --- | --- | --- | --- | --- | --- | --- |
| Drug concentration  (ng /ml) | Pi | 0 | 0 | 0 | 40 | 40 | 0 | 0 | 0 | 0 |
|  | Pa | 0 | 0 | 160 | 0 | 0 | 160 | 0 | 0 | 0 |
|  | EH | 0 | 250 | 0 | 0 | 0 | 0 | 250 | 0 | 0 |
|  | Ci | 1500 | 1500 | 1500 | 1500 | 0 | 0 | 0 | 1500 | 0 |
|  | Mi | 200 | 200 | 200 | 200 | 0 | 0 | 0 | 0 | 200 |
| Cell survival  (%) | 5637 | 18.3 | 1.06 | 7.8 | 4.36 | 29.22 | 6.45 | 14.75 | 20.32 | 53.16 |
|  | Biu87 | 13.7 | 12.5 | 8.5 | 12.3 | 41.67 | 23.08 | 38.68 | 20.94 | 42.05 |
|  | J82 | 17.9 | 7.26 | 11.8 | 7.7 | 55.37 | 38.51 | 45.03 | 15.42 | 48.38 |
|  | T24 | 15.5 | 5.82 | 6.73 | 10.4 | 48.33 | 11.55 | 20.13 | 15.46 | 51.34 |
|  | EJ | 24.85 | 17.14 | 18.32 | 18.01 | 30.38 | 33.05 | 56.55 | 32.34 | 45.2 |
|  | UM-UC-3 | 13.1 | 4.2 | 7.02 | 8.86 | 65.59 | 40.09 | 43.54 | 14.71 | 66.2 |
|  | H-bc | 43.74 | 0.48 | 34.73 | 5.93 | 86.89 | 13.96 | 61.03 | 41.98 | 91.67 |
|  | SCaBER | 17.63 | 3.01 | 14.22 | 3.49 | 30.04 | 57.91 | 38.31 | 22.05 | 56.83 |
|  | ACS (%) | 20.59 | 6.43 | 13.64 | 8.88 | 48.44 | 28.08 | 39.75 | 22.9 | 56.85 |

**Table S4. Further experimental testing of the EH/Ci/Mi regimen (1).** The tri-drug combinations (1-4) and five drug at a one fold dose (ng/mL) are indicated and the cell survival (%) of each treated cell line were measured, from which the ACS (%) of each combination was calculated (**A**).

| **5637** | **Mock** | | | **Ci** | | | **Mi** | | | **EH** | | | **EH/Ci/Mi** | | |
| --- | --- | --- | --- | --- | --- | --- | --- | --- | --- | --- | --- | --- | --- | --- | --- |
|  | **luc** | **Renluc** | **Relative activity** | **luc** | **Renluc** | **Relative activity** | **luc** | **Renluc** | **Relative activity** | **luc** | **Renluc** | **Relative activity** | **luc** | **Renluc** | **Relative activity** |
| **DNA Damage** | 12536 | 29072830 | 0.22 | 16463 | 30579432 | 0.48 | 17956 | 34938044 | 0.63 | 16120 | 30098156 | 0.75 | 15629 | 26196576 | 0.83 |
| **Hypoxia** | 1052057 | 23600716 | 22.72 | 1024324 | 24274040 | 37.36 | 1352097 | 22097140 | 75.18 | 808185 | 22038922 | 51.46 | 906153 | 22826258 | 54.96 |
| **ER Stress** | 1601415 | 31382872 | 26.01 | 1662012 | 31011830 | 47.45 | 2175983 | 32174418 | 83.09 | 1698175 | 28112488 | 84.76 | 2088434 | 30298250 | 95.44 |
| **Heat Shock** | 511659 | 18502666 | 14.09 | 256314 | 24711858 | 9.18 | 423550 | 26726118 | 19.47 | 241002 | 23164298 | 14.60 | 433669 | 22347410 | 26.87 |
| **Wnt** | 47316 | 27569552 | 0.87 | 29940 | 26540942 | 1.00 | 36165 | 27194420 | 1.63 | 19535 | 26427688 | 1.04 | 20032 | 22864232 | 1.21 |
| **Notch** | 42575 | 21358194 | 1.02 | 36939 | 23115178 | 1.41 | 46653 | 25672570 | 2.23 | 36211 | 25995968 | 1.95 | 55387 | 20590916 | 3.72 |
| **Cell Cycle/pRb-E2F** | 90588 | 50884692 | 0.91 | 118387 | 59247780 | 1.77 | 122055 | 62526588 | 2.40 | 214150 | 64347208 | 4.67 | 141777 | 56925468 | 3.45 |
| **Myc/Max** | 634048 | 29187098 | 11.07 | 368299 | 28010318 | 11.64 | 475193 | 26813652 | 21.77 | 318318 | 26899256 | 16.61 | 479680 | 24095642 | 27.56 |
| **MAPK/ERK** | 2257440 | 22080312 | 52.10 | 2070782 | 20198954 | 90.77 | 2811788 | 17753446 | 194.59 | 1226693 | 14625057 | 117.70 | 1764958 | 15854674 | 154.13 |
| **Negative control** | 46553 | 23724400 | 1 | 29773 | 26360576 | 1 | 19188 | 23574666 | 1 | 14253 | 20000002 | 1 | 15134 | 20953624 | 1 |
| **Positive control** | 2827637 | 17096506 | 84.29 | 3033778 | 13923928 | 192.91 | 2465498 | 16347385 | 185.30 | 1537250 | 12755471 | 169.11 | 2013196 | 15850561 | 175.85 |

**Table S5. The signaling pathway analysis in 5637.** The transfection of cells was performed in duplicate. The cell lysate from two transfected cells of the pair were combined for the activities of both firefly luciferase and *renilla* luciferase genes, which are listed as luc and Renluc in each treatment. Background relative luciferase activities are listed as Mock. Luciferase activities of the EH/Ci/Mi combination versus that by a 4 fold of the single drug: Ci, Mi, and EH are listed in the table.

| **Biu87** | **Mock** | | | **Ci** | | | **Mi** | | | **EH** | | | **EH/Ci/Mi** | | |
| --- | --- | --- | --- | --- | --- | --- | --- | --- | --- | --- | --- | --- | --- | --- | --- |
|  | **luc** | **Renluc** | **Relative activity** | **Luc** | **Renluc** | **Relative activity** | **luc** | **Renluc** | **Relative activity** | **luc** | **Renluc** | **Relative activity** | **luc** | **Renluc** | **Relative activity** |
| **DNA Damage** | 1170 | 2227384 | 1.51 | 1663 | 3531196 | 0.94 | 2890 | 5710684 | 2.03 | 1470 | 2834282 | 1.58 | 1371 | 5077079 | 1.08 |
| **Hypoxia** | 4451 | 1687236 | 7.58 | 7487 | 1499278 | 10.01 | 7691 | 1144978 | 26.99 | 6519 | 1156255 | 17.13 | 9376 | 936424 | 40.22 |
| **ER Stress** | 40626 | 5327666 | 21.92 | 74981 | 8302932 | 18.10 | 44361 | 4931940 | 36.14 | 52055 | 4349360 | 36.36 | 57183 | 4678703 | 49.10 |
| **Heat Shock** | 3389 | 5145078 | 1.89 | 9053 | 6321098 | 2.87 | 8222 | 3842602 | 8.60 | 3274 | 4953542 | 2.01 | 2263 | 3712893 | 2.45 |
| **Wnt** | 1084 | 1873321 | 1.66 | 1035 | 3005559 | 0.69 | 1100 | 2497907 | 1.77 | 1274 | 2458774 | 1.57 | 852 | 3350532 | 1.02 |
| **Notch** | 3188 | 2067691 | 4.43 | 4447 | 3994370 | 2.23 | 4045 | 2530636 | 6.42 | 4323 | 2210120 | 5.94 | 5131 | 2737670 | 7.53 |
| **Cell Cycle/pRb-E2F** | 1048 | 5308695 | 0.57 | 827 | 5334082 | 0.31 | 792 | 3836588 | 0.83 | 3978 | 5391695 | 2.24 | 751 | 6701679 | 0.45 |
| **Myc/Max** | 6849 | 2405994 | 8.18 | 10012 | 4361355 | 4.60 | 9217 | 3901945 | 9.49 | 13337 | 2976075 | 13.61 | 14966 | 3270559 | 18.38 |
| **MAPK/ERK** | 219738 | 1289376 | 489.80 | 223810 | 1396421 | 321.19 | 454932 | 1824789 | 1001.57 | 402732 | 2011157 | 608.33 | 210769 | 1578983 | 536.23 |
| **Negative control** | 2475 | 7113303 | 1 | 3696 | 7406714 | 1 | 2398 | 9633820 | 1 | 2575 | 7822520 | 1 | 2087 | 8383868 | 1 |
| **Positive control** | 111213 | 1913179 | 167.07 | 137362 | 1480354 | 185.95 | 123326 | 1627869 | 304.36 | 156600 | 1862408 | 255.44 | 194031 | 1520161 | 512.75 |

**Table S6. The signaling pathway analysis in Biu87**. The transfection of cells was performed in duplicate. The cell lysate from two transfected cells of the pair were combined for the activities of both firefly luciferase and *renilla* luciferase genes, which are listed as luc and Renluc in each treatment. Background relative luciferase activities are listed as Mock. Luciferase activities of the EH/Ci/Mi combination versus that by a 4 fold of the single drug: Ci, Mi, and EH are listed in the table.

| **UM-UC-3** | **Mock** | | | **Ci** | | | **Mi** | | | **EH** | | | **EH/Ci/Mi** | | |
| --- | --- | --- | --- | --- | --- | --- | --- | --- | --- | --- | --- | --- | --- | --- | --- |
|  | **luc** | **Renluc** | **Relative activity** | **luc** | **Renluc** | **Relative activity** | **luc** | **Renluc** | **Relative activity** | **luc** | **Renluc** | **Relative activity** | **luc** | **Renluc** | **Relative activity** |
| **DNA Damage** | 5855 | 1472476 | 3.49 | 5325 | 2147032 | 2.38 | 8237 | 1502130 | 4.77 | 9467 | 1431776 | 5.96 | 8320 | 1524256 | 5.42 |
| **Hypoxia** | 33444 | 468141 | 62.72 | 25797 | 594608 | 41.68 | 29511 | 558774 | 45.95 | 39411 | 480634 | 73.93 | 29518 | 506501 | 57.87 |
| **ER Stress** | 91342 | 982640 | 81.61 | 71599 | 1019011 | 67.50 | 81715 | 648793 | 109.59 | 114029 | 849848 | 120.98 | 63215 | 948602 | 66.17 |
| **Heat Shock** | 929 | 187527 | 4.35 | 550 | 154516 | 3.42 | 1002 | 216089 | 4.03 | 536 | 164788 | 2.93 | 338 | 160863 | 2.09 |
| **Wnt** | 2272 | 555610 | 3.59 | 1099 | 610446 | 1.73 | 2244 | 480824 | 4.06 | 2599 | 568504 | 4.12 | 1253 | 430152 | 2.89 |
| **Notch** | 23352 | 4144667 | 4.95 | 20903 | 4021789 | 4.99 | 22993 | 2702600 | 7.40 | 34486 | 3060547 | 10.16 | 26857 | 3559553 | 7.49 |
| **Cell Cycle/pRb-E2F** | 1230 | 181856 | 5.94 | 561 | 234963 | 2.29 | 787 | 315046 | 2.17 | 3739 | 195419 | 17.25 | 1292 | 215829 | 5.94 |
| **Myc/Max** | 21009 | 316030 | 58.37 | 11423 | 449464 | 24.42 | 11805 | 458417 | 22.41 | 15832 | 560204 | 25.48 | 12508 | 303171 | 40.97 |
| **MAPK/ERK** | 93465 | 576801 | 142.27 | 54365 | 724900 | 72.05 | 96470 | 554587 | 151.35 | 134413 | 661484 | 183.22 | 70680 | 644165 | 108.95 |
| **Negative control** | 11172 | 9808636 | 1 | 10236 | 9833868 | 1 | 10178 | 8855745 | 1 | 10475 | 9444867 | 1 | 9389 | 9322670 | 1 |
| **Positive control** | 309127 | 2534228 | 107.09 | 266403 | 2002668 | 127.80 | 219764 | 2347988 | 81.44 | 328077 | 2808991 | 105.31 | 319831 | 2468161 | 128.67 |

**Table S7. The signaling pathway analysis in UM-UC-3.** The transfection of cells was performed in duplicate. The cell lysate from two transfected cells of the pair were combined for the activities of both firefly luciferase and *renilla* luciferase genes, which are listed as luc and Renluc in each treatment. Background relative luciferase activities are listed as Mock. Luciferase activities of the EH/Ci/Mi combination versus that by a 4 fold of the single drug: Ci, Mi, and EH are listed in the table.

| **H-bc** | **Mock** | | | **Ci** | | | **Mi** | | | **EH** | | | **EH/Ci/Mi** | | |
| --- | --- | --- | --- | --- | --- | --- | --- | --- | --- | --- | --- | --- | --- | --- | --- |
|  | **luc** | **Renluc** | **Relative activity** | **luc** | **Renluc** | **Relative activity** | **luc** | **Renluc** | **Relative activity** | **luc** | **Renluc** | **Relative activity** | **luc** | **Renluc** | **Relative activity** |
| **DNA Damage** | 2015 | 3432545 | 0.80 | 1546 | 1281912 | 4.19 | 1185 | 1990956 | 0.73 | 2158 | 692083 | 3.99 | 2227 | 1073909 | 1.47 |
| **Hypoxia** | 93683 | 3105480 | 41.20 | 106095 | 7500511 | 49.11 | 108144 | 6568317 | 20.08 | 128282 | 2852609 | 57.60 | 151139 | 6899389 | 15.49 |
| **ER Stress** | 94542 | 4743843 | 27.22 | 163717 | 5594167 | 101.61 | 239204 | 6560323 | 44.48 | 181107 | 8106904 | 28.61 | 223897 | 10687862 | 14.81 |
| **Heat Shock** | 4692 | 1440244 | 4.45 | 8865 | 1886689 | 16.31 | 4846 | 5282890 | 1.12 | 4438 | 1818590 | 3.13 | 4225 | 1753539 | 1.70 |
| **Wnt** | 4284 | 4474023 | 1.31 | 5450 | 6023579 | 3.14 | 5492 | 11151935 | 0.60 | 7571 | 8824576 | 1.10 | 6611 | 6578338 | 0.71 |
| **Notch** | 1607 | 1298732 | 1.69 | 2219 | 4825030 | 1.60 | 1832 | 3790065 | 0.59 | 2986 | 1741009 | 2.20 | 2697 | 3162138 | 0.60 |
| **Cell Cycle/pRb-E2F** | 10459 | 3833178 | 3.73 | 11455 | 6792549 | 5.86 | 12829 | 17003119 | 0.92 | 17368 | 5010011 | 4.44 | 12065 | 4671758 | 1.83 |
| **Myc/Max** | 89185 | 7685777 | 15.85 | 120068 | 8299990 | 50.23 | 86926 | 10750527 | 9.86 | 92499 | 6723079 | 17.62 | 86003 | 2918818 | 20.84 |
| **MAPK/ERK** | 239072 | 2086866 | 156.44 | 290167 | 2958210 | 340.57 | 325601 | 6723697 | 59.07 | 331906 | 4467438 | 95.16 | 393552 | 5160822 | 53.92 |
| **Negative control** | 703 | 959998 | 1 | 954 | 3312384 | 1 | 914 | 1114948 | 1 | 838 | 1073349 | 1 | 724 | 511956 | 1 |
| **Positive control** | 22609 | 2499255 | 12.35 | 14602 | 2857566 | 17.74 | 18615 | 6370362 | 3.56 | 13915 | 2479590 | 7.19 | 20173 | 1489971 | 9.57 |

**Table S8. The signaling pathway analysis in H-bc.** The transfection of cells was performed in duplicate. The cell lysate from two transfected cells of the pair were combined for the activities of both firefly luciferase and *renilla* luciferase genes, which are listed as luc and Renluc in each treatment. Background relative luciferase activities are listed as Mock. Luciferase activities of the EH/Ci/Mi combination versus that by a 4 fold of the single drug: Ci, Mi, and EH are listed in the table.

| **SV-HUC-1** | **Mock** | | | **Ci** | | | **Mi** | | | **EH** | | | **EH/Ci/Mi** | | |
| --- | --- | --- | --- | --- | --- | --- | --- | --- | --- | --- | --- | --- | --- | --- | --- |
|  | **luc** | **Renluc** | **Relative activity** | **luc** | **Renluc** | **Relative activity** | **luc** | **Renluc** | **Relative activity** | **luc** | **Renluc** | **Relative activity** | **luc** | **Renluc** | **Relative activity** |
| **DNA Damage** | 65065 | 26021553 | 2.40 | 67503 | 24632906 | 3.70 | 281508 | 31469814 | 4.20 | 310089 | 31955895 | 6.00 | 389590 | 26547677 | 5.60 |
| **Hypoxia** | 556794 | 21123753 | 25.30 | 437361 | 19553671 | 30.20 | 2225553 | 19903601 | 52.50 | 1665093 | 23399224 | 44.00 | 2546002 | 23132188 | 42.00 |
| **ER Stress** | 2999614 | 28089149 | 102.50 | 2127729 | 24981219 | 115.00 | 9579549 | 28980528 | 155.20 | 14481584 | 29847666 | 300.00 | 12552116 | 30704324 | 156.00 |
| **Heat Shock** | 683248 | 16560758 | 39.60 | 542556 | 19906350 | 36.80 | 2050872 | 24073070 | 40.00 | 1431915 | 24594061 | 36.00 | 2492592 | 22646922 | 42.00 |
| **Wnt** | 31879 | 24676048 | 1.24 | 24544 | 21379748 | 1.55 | 83472 | 24494884 | 1.60 | 58993 | 28058876 | 1.30 | 91080 | 23170671 | 1.50 |
| **Notch** | 85641 | 19116590 | 4.30 | 85503 | 18620163 | 6.20 | 201928 | 23124105 | 4.10 | 249971 | 27600509 | 5.60 | 278882 | 20866887 | 5.10 |
| **Cell Cycle/pRb-E2F** | 232505 | 45544197 | 4.90 | 296922 | 47726361 | 8.40 | 815673 | 56319698 | 6.80 | 1325887 | 68318891 | 12.00 | 1058228 | 57688414 | 7.00 |
| **Myc/Max** | 2999310 | 26123828 | 110.20 | 1833224 | 22563387 | 109.70 | 8230355 | 24151914 | 160.00 | 5773588 | 28559550 | 125.00 | 6399018 | 24418585 | 100.00 |
| **MAPK/ERK** | 4857155 | 19762919 | 235.90 | 4687804 | 16271033 | 389.00 | 11409592 | 15991097 | 335.00 | 5650354 | 15527755 | 225.00 | 10105164 | 16067167 | 240.00 |
| **Negative control** | 22123 | 21234456 | 1.00 | 15727 | 21234456 | 1.00 | 45226 | 21234456 | 1.00 | 34342 | 21234456 | 1.00 | 55646 | 21234456 | 1.00 |
| **Positive control** | 1992811 | 15302179 | 125.00 | 1287610 | 11216259 | 155.00 | 6679909 | 14724613 | 213.00 | 3854825 | 13542773 | 176.00 | 7450625 | 16062999 | 177.00 |

**Table S9. The signaling pathway analysis in** **SV-HUC-1.** The transfection of cells was performed in duplicate. The cell lysate from two transfected cells of the pair were combined for the activities of both firefly luciferase and *renilla* luciferase genes, which are listed as luc and Renluc in each treatment. Background relative luciferase activities are listed as Mock. Luciferase activities of the EH/Ci/Mi combination versus that by a 4 fold of the single drug: Ci, Mi, and EH are listed in the table.

**Supplementary Materials 2: Differential Evolution search algorithm used in this study**

There are three calculations steps in the DE search algorithm: “Mutation”, “Crossover” and “Selection” calculations, used together to suggest the list of the options to be tested in the remaining three round screenings (Fig. 1).

The drug combination in the trial group was calculated, according to the mathematical equation below:
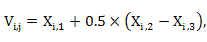
 where
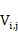
 represents a “Mutation” calculated drug combination,
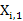
,
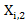
,
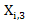
are different random drug combinations from the trial group. This process was repeated twenty times to generate twenty “Mutation” drug combinations, which were tested in cell lines for their treatment-trigged cell death (iteration 1). The next calculation is called “Crossover” calculation, which is based on the following set of mathematical equations:
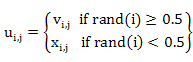
, where
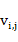
 and
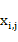
 means the drug and dose in “Mutation” drug combinations and trial group, respectively,
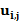
 means the drug and dose in the “Crossover” calculated drug combinations. This calculation was repeated for each drug in the “Mutation” drug combinations to generate twenty “Crossover” drug combinations (also named the test group,) (Step 2), which were experimentally tested (iteration 2). In the “Selection” calculation, the drug-triggered cell death of trial groups (iteration 1 and 2 were compared, based on the following calculation:
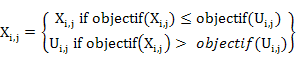
, where X_i,j_ represents the drug combinations in the trial group and U_i,j_ represents the drug combinations in the test group. Drug combinations with better efficacy were left for further consideration via the same calculation process to again generate a new test group.

**Supplementary Materials 3: Analysis of statistical modeling of four cell lines to decide component drugs for tri-drug combination**

After four rounds of drug combination optimization experiments under the guidance of the Feedback System Control (FSC) approach, the linear regression statistical modeling for the following four cell lines was built: Biu87, H-bc, 5637, and UM-UC-3

The linear regression model for Biu87 was:


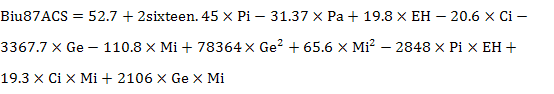
, where Biu87ACS represents the average of accumulated cell survival (%) of Biu87 cells under drug treatment. Pi, Pa, EH, Ci, Ge, and Mi in the equation represent the absolute dosages of each drug (ng/mL). The model had a R^2^ value equals to 0.7015 and p-value equals to 2.439e-15, which indicated a fairly good fit between the model and real experimental observations. Ge had a negative linear regression coefficient but largest positive quadratic regression coefficient, which means the dose increase of Ge is less likely to positively contribute to the drug-triggered cell death when it is used in combination with other drugs. Ge also had the largest positive two-drug interaction coefficient with Mi, meaning in the presence of Mi, Ge could have an antagonistic effect in reducing cell survival. Therefore, we confidently eliminated Ge from further study.

The regression model for the H-bc cell line was:
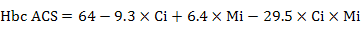


For the H-bc cell line, only Ci and Mi were left in the final regression model and the rest of the drugs did not appear in the model. The interaction coefficient was negative, suggesting a synergistic effect could exist between the two drugs in treating the H-bc cell line, indicating that Ci and Mi were the most dominant drugs in treating the H-bc cell line.

The model for cell line 5637 was :


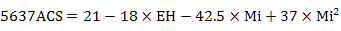
;

For cell line 5637, only EH and Mi were left in the final regression model, suggesting EH and Mi were the most effective drugs in treating cell line 5637.

The model for the UM-UC-3 cell line was:

UM-UC-3 ACS
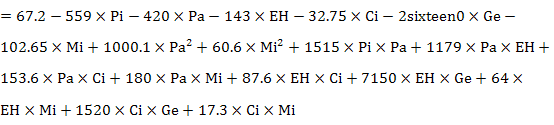


For the UM-UC-3 cell line, all six drugs showed negative coefficients, meaning as a single drug, they all should contribute to the reduction of UM-UC-3 growth. However, the interaction coefficients were all positive, which indicated that when accumulative drug doses start to increase, especially when the drug doses are high enough, the contribution of drug synergy are not clearly observed in the mixture of the six drugs. Another way to interpret this is that when treating UM-UC-3, a subgroup of the six drugs may be no worse than the combination of the six drugs. Furthermore, Pa and Pi showed a very large positive interaction coefficient, Pa also showed a very large quadratic coefficient, so Pa and Pi were used in combination for the UM-UC-3 cell line. Since EH, Ci, and Mi were most commonly seen drugs in all the models, based on the statistical analysis discussed above, a tri-drug combination EH/Ci/Mi was confirmed for further investigation in our study.

**Supplementary Materials 4. Use of Feedback System Control (FSC) Platform in the Present Study**

Feedback System Control (FSC) is a platform technology for rapid identification of optimal drug combinations. FSC consists of three iterative operations: 1) suggesting candidate drug combinations, 2) acquiring readouts of each combination experimentally, and 3) optimizing the combinations through an engineering feedback system control algorithm (*1, 2*).

Compared to the conventional experimental setups where high-throughput tests are often included, the FSC approach tests only a small number of drug combinations (usually less than 25) per iteration and optimizes them iteratively. Usually, the optimal solution for the system can be identified in no more than 20 iterations by testing 0.1~1% of the total possibilities. The FSC technique has been verified in multiple systems, including viral inhibition (*1*), human ES cell maintenance (*3*), and Chinese herbal medicine (*4*). In this project, we aimed to apply the FSC strategy to optimize 8 chemo-drugs for 8 cell lines. An illustrative scheme is shown in Fig. 1.

The success of the FSC scheme highly relies on system readouts that closely imitate the ideal biological activity. The readouts should fairly indicate the efficiency of the therapy, in our case, the suitable and differential lethal rate for different cells. The potent drug combination(s) that could kill most cancer cells with the least side effect on the normal cells was our goal.

In order to decide on a reasonable dose range of each drug to be studied, the IC_50_ profiles of all 8 cell lines were first established. Each drug was designated with five doses which differed by 5 fold to cover most of the range from 0 up to IC_50_ dose.

The first batch of drug combinations to be tested were randomly generated through a random number generator written in MATLAB language (MathWorks©, Natick, MA, U.S.).

The engineering feedback algorithm in this study was Differential Evolution, a robust search algorithm that had previously been verified in a viral inhibition system(*1*).

**Reference**

1. X. Ding, D. J. Sanchez, A. Shahangian, I. Al-Shyoukh, G. Cheng, C. M. Ho, Cascade search for HSV-1 combinatorial drugs with high antiviral efficacy and low toxicity. *International journal of nanomedicine***7**, 2281-2292 (2012).

2.P. K. Wong, F. Yu, A. Shahangian, G. Cheng, R. Sun, C. M. Ho, Closed-loop control of cellular functions using combinatory drugs guided by a stochastic search algorithm. *Proceedings of the National Academy of Sciences of the United States of America***105**, 5105-5110 (2008).

3.H. Tsutsui, B. Valamehr, A. Hindoyan, R. Qiao, X. Ding, S. Guo, O. N. Witte, X. Liu, C. M. Ho, H. Wu, An optimized small molecule inhibitor cocktail supports long-term maintenance of human embryonic stem cells. *Nature communications***2**, sixteen7 (2011).

4.H. Yu, W. L. Zhang, X. Ding, K. Y. Zheng, C. M. Ho, K. W. Tsim, Y. K. Lee, Optimizing combinations of flavonoids deriving from astragali radix in activating the regulatory element of erythropoietin by a feedback system control scheme. *Evidence-based complementary and alternative medicine : eCAM***2013**, 541436 (2013).
